# Exercise training partly ameliorates cardiac dysfunction in mice during doxorubicin treatment of breast cancer

**DOI:** 10.1186/s12967-025-06108-y

**Published:** 2025-01-21

**Authors:** Tytti-Maria Uurasmaa, Pauline Bourdin, Wail Nammas, Shiva Latifi, Heidi Liljenbäck, Antti Saraste, Olli Eskola, Johan Rajander, Anne Roivainen, Helene Rundqvist, Anu Autio, Ilkka Heinonen, Katja Anttila

**Affiliations:** 1 Department of Biology, University of Turku, Turku, Finland; 2 Turku PET Centre, University of Turku, Turku University Hospital, Turku, Finland; 3 Turku Center for Disease Modeling, University of Turku, Turku, Finland; 4 Heart Centre, Turku University Hospital and University of Turku, Turku, Finland; 5 Accelerator Laboratory, Åbo Akademi University, Turku, Finland; 6 InFLAMES Research Flagship, University of Turku, Turku, Finland; 7 Department of Laboratory Medicine, Karolinska Institute, Stockholm, Sweden

**Keywords:** Glucose metabolism, Cardiotoxicity, Exercise, Anthracyclines, CS, LDH

## Abstract

**Introduction:**

Doxorubicin is a chemotherapeutic drug used to treat various cancers. Exercise training (ET) can attenuate some cardiotoxic effects of doxorubicin (DOX) in tumor-free animals. However, the ET effects on cardiac function and glucose metabolism in DOX-treated breast cancer models remain unclear.

**Objectives:**

This study investigated ET-induced structural, functional, vascular, oxidative stress, and plausible glucose uptake alterations of the left ventricle (LV) in a murine breast cancer model during DOX treatment.

**Methods:**

Female FVB/N-mice were divided to tumor-free groups with or without voluntary wheel-running ET and those inoculated subcutaneously with mammary tumor-derived I3TC-cells with or without exercise or DOX treatment (5 mg/kg/week). Mice underwent 2-[^18^F]fluoro-2-deoxy-D-glucose positron emission tomography and echocardiography after two and four DOX-doses. The cardiac histology, oxidative stress, maximal metabolic enzyme activities, and mitochondrial respiration were analyzed.

**Results:**

DOX increased LV glucose uptake (LVGU) and mitochondrial uncoupling and decreased running activity, LV-weight, and ejection fraction (EF). In DOX-treated group ET blunted the increase in LVGU, increased LV-weight and EF, and lowered LV lactate dehydrogenase activity. Exercised mice had lower LVGU compared to unexercised groups and DOX-treated ET-group did not differ from tumor-free ET-group in LV-weight or EF whereas unexercised DOX-treated group did. ET also increased LV citrate synthase activity in tumor-bearing animals. There was an inverse association between LVGU and EF and LV-weight.

**Conclusion:**

In a murine breast cancer model, voluntary ET moderated DOX-induced cardiotoxicities such as increased LVGU, LV-atrophy and decreased EF. This suggests that ET might benefit patients with cancer undergoing doxorubicin treatment by mitigating cardiotoxicity.

**Supplementary Information:**

The online version contains supplementary material available at 10.1186/s12967-025-06108-y.

## Introduction

Anthracyclines have long been used to treat cancers such as breast cancer, leukemia, and lymphoma due to their effectiveness against tumors cells, however they can cause cardiotoxicity. Currently not many pharmacological interventions exist for prevention of anthracycline-induced cardiotoxicity, with Dexrazoxane being the only U.S. FDA approved drug for the purpose [1]. The cardiotoxic effects of anthracycline doxorubicin (DOX) limit its use and therefore investigations to mitigate its toxicity are needed. Many preclinical studies and some clinical studies have shown that exercise training (ET) can reduce DOX-induced cardiotoxicity, but majority of preclinical studies utilize tumor-free models and ET preconditioning [2–4]. There is a lack of ET intervention studies utilizing tumor-bearing cardiotoxicity models and investigating alterations in glucose metabolism [4]. Glucose metabolism being interesting in this context because there is increasing evidence of DOX altering cardiac metabolism [10]. Therefore, in the present study we comprehensively investigated possible alterations in cardiac structure and function such as glucose uptake to explore if ET could mitigate the cardiotoxic effects of DOX-dosing during treatment in mammary tumor-bearing mice.

ET has already been shown to improve at least the skeletal muscle glucose metabolism in tumor bearing animals [5] and there is evidence that exercise could be beneficial therapeutic tool in the clinical setting, affecting particularly the skeletal muscle but also possibly the heart [6, 7]. ET has been shown to reduce mitochondrial DOX-accumulation and reduce DOX-induced cardiac dysfunction and in some cases cardiac oxidative stress by increasing antioxidative enzyme activities [4, 8, 9]. A review by Russo et al. (2021), however, addressed the need for more studies focusing on DOX-induced metabolic alterations since antioxidant treatments have been unable to effectively counter anthracycline-induced cardiotoxicity, suggesting significant contribution of other mechanisms besides oxidative stress [10]. Indeed, drugs used to treat metabolic diseases, such as sodium-glucose co-transporter 2 inhibitors, have had promising effects regarding reduction of anthracycline-induced cardiotoxicity [10].

DOX increases cardiac glucose uptake (GU) as measured by glucose analog 2-[^18^F]fluoro-2-deoxy-*D*-glucose (FDG) [11–14], and it has been theorized that this could be a compensatory mechanism to improve cardiac metabolism or maintain NADPH-reserves for glutathione antioxidative function in response to DOX-induced oxidative stress [12]. However, to our knowledge the effects of ET on cardiac GU during DOX treatment of breast cancer have not been studied earlier.

Consequently, the main aims of this study were to investigate comprehensively the effects of DOX on the cardiac GU and maximal metabolic enzyme activities, LV structure and function and vasculature, oxidative stress as well as how voluntary wheel-running ET could influence these variables during DOX treatment in a murine breast cancer model.

## Materials and methods

Detailed materials and methods are provided in supplementary material Additional File 1.

### Animals and the experimental protocol

The animal studies were approved by the national Project Authorization Board (permission number ESAVI/26508/2021) and were carried out in compliance with the EU Directive 2010/EU/63 on the protection of animals used for scientific purposes. Ten-week-old female FVB/NHan^®^Hsd mice (21 ± 1.5 g) were randomly divided into six groups: tumor-free control (C, *n* = 10); tumor-free control with exercise (CE, *n* = 12); I3TC-tumor group (T, *n* = 15); I3TC-tumor group with exercise (TE, *n* = 13); I3TC-tumor group with DOX treatment (TD, *n* = 12); I3TC-tumor group with DOX treatment and exercise (TDE, *n* = 12). Power calculation was not done as this was an explorative study, the sample size was chosen according to review of mouse studies on exercise effects on DOX induced cardiotoxicity, majority of which had sample size of 7–13 animals [3].

**Fig. 1:**
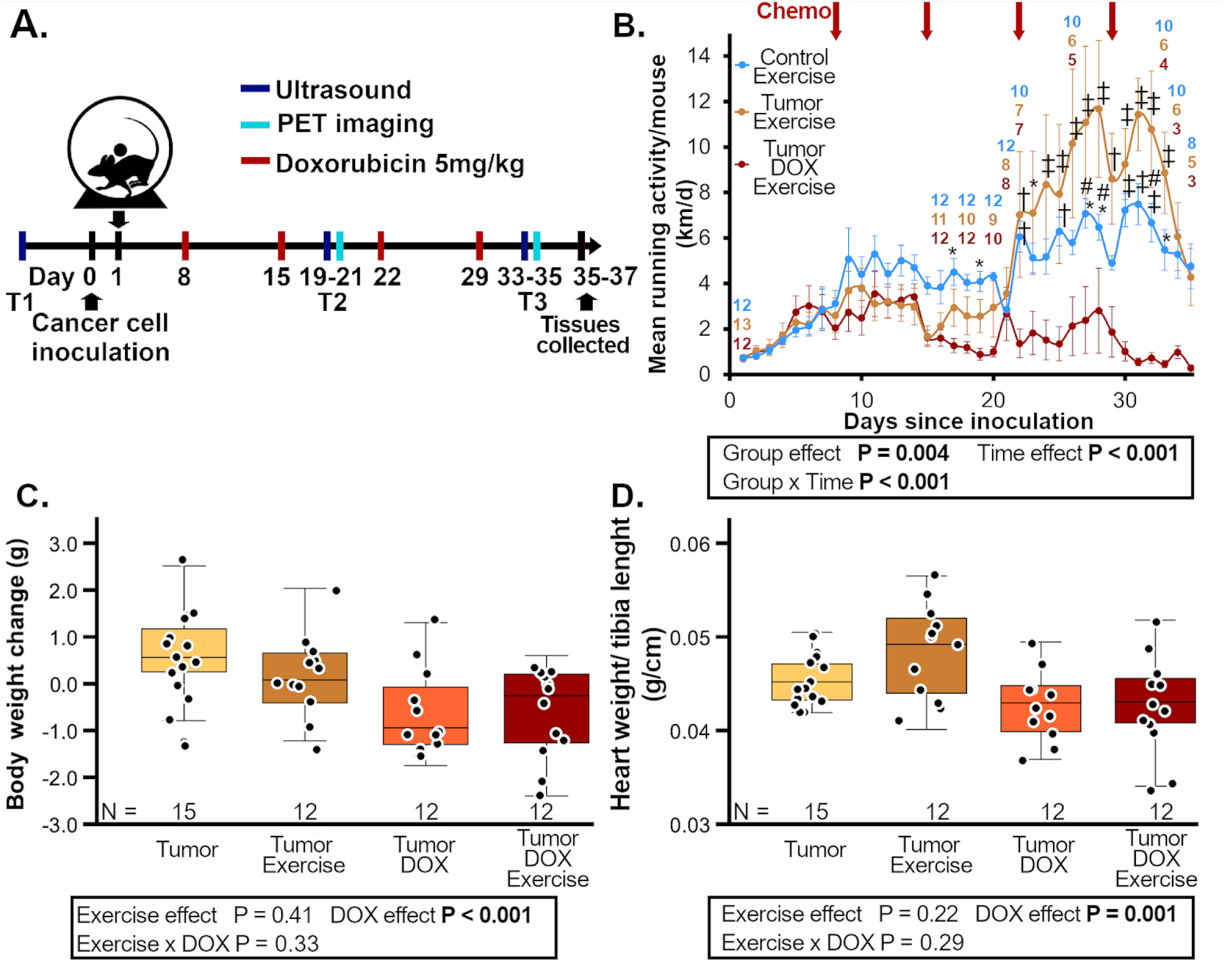
(**A**) Study-timeline, (**B**) wheel-running ± SE, (**C**) body weights and (**D**) heart weights. Groups are female FVB-mice with or without subcutaneous I3TC-tumors or DOX treatment with group n-numbers (N) indicated overtime in group colors or on the x-axis below each group boxplot. Two-way ANOVA *P* < 0.05 are highlighted in bold. Holm-Sidak *P < 0.05 and †*P* < 0.01 and ‡*P* ≤ 0.001 versus Tumor-DOX-Exercise and #*P* < 0.05 versus Tumor-Exercise. The data obtained for current manuscript in C and D from the tumor group without exercise and without doxorubicin treatment has been previously published in Koivula et al. (2024) to support clinical findings [36]

Day after subcutaneous inoculation of 1.8 × 10⁶ I3TC-cells [15, 16] or phosphate-buffered saline (PBS) to the flank, the animals (2/cage) were given running wheels (locked for no-exercise) (Fig. 1A). Eight days later intraperitoneally administered DOX or PBS treatment was started (5 mg/kg/week). The animals were euthanized, and organs were collected a week after the fourth DOX-dose or earlier if the tumor diameter reached 1.5 cm. Mice in the same cage were assumed to run equally, possibly underestimating running activity since mice can also use the wheel simultaneously.

### Echocardiography

All mice underwent transthoracic echocardiography at baseline (T1), 2.8 weeks (T2) and 4.8 weeks after tumor cell inoculation (T3). Heart rate (HR), ejection fraction (EF), left ventricle mass (LVM), LV outflow tract velocity time integral (LVOT VTI) and mitral early diastolic (E) and late diastolic (A) blood velocity ratio (E/A) were calculated.

### PET/CT-imaging

A random subset of mice was imaged with PET/CT at T2 and T3. The LV standardized FDG-uptake values (FDG-SUVs) normalized for the injected radioactivity dose and animal body weight as a measure for LVGU were calculated using Carimas-software [17].

### Mitochondrial respiration from tissue homogenate

The mitochondrial respiration of LV homogenates, which tells about the mitochondrial oxygen consumption rate, was measured with high-resolution respirometry oxygraph 2k [18]. The substrates/inhibitors were added sequentially to assess oxygen fluxes corresponding to uncoupled proton leak (LEAK), complex 1–2 linked oxidative phosphorylation, maximal electron transfer capacity, complex 2 linked electron transfer and maximal complex 4 activity. Mitochondrial respirations were normalized in three different ways respectively; per tissue mass, citrate synthase activity and mitochondrial index measured using qPCR. The coupling efficiency was calculated by complex 1 driven coupled respiration divided by complex 1 linked total respiration. Additionally, CS/CIV-ratio was calculated by maximal citrate synthase (CS) activity divided by complex 4 linked maximal respiration, both per tissue mass. Normalization per tissue mass revealing tissue level oxygen consumption rate and the citrate synthase activity and mitochondrial index normalization revealing oxygen consumption rate in relation to mitochondrial quantity assessed in different ways.

### Histology

Average LV capillary density and cell numbers were calculated from transversal paraffin-embedded sections stained with Periodic Acid-Schiff Stain [19].

### Enzymatic assays and oxidative stress measurements

The mouse LVs were homogenized for spectrophotometric measurement of lipid peroxidation (LPX) and protein carbonylation (CARB) and the maximal activities of 3-hydroxyacyl-CoA dehydrogenase (HOAD), CS, lactate dehydrogenase (LDH), superoxide dismutase and catalase. Gastrocnemius LDH, CS and LPX were also measured. Outliers > 2 × SD were excluded from analyses due to the possibility of methodological outliers.

### Statistical analysis

Single time point parameters were compared with two-way ANOVA to analyze DOX and ET effects within cancer groups (comparison between T, TE, TD, TDE) and to analyze cancer and ET effects within no-DOX groups (comparisons between C, CE, T, TE).

The running activity between ET groups was compared using two-way RM ANOVA with time and group as factors. The other repeated measure parameters were compared between all the groups over time using proc Glimmix linear mixed model on repeated measures with unstructured covariance. All groups were compared together using exercise, time point, and chemotherapy nested under breast cancer as the factors. The effect of cancer alone was tested separately comparing the no-DOX groups using breast cancer, exercise and time point as factors. Spearman correlation of LV FDG-SUV with EF and LVM at T2 and T3 was analyzed as well as correlation of T3 EF with mitochondrial coupling, LEAK and LVM.

For all group comparisons Holm-Sidak post-hoc test was performed if ANOVA or linear mixed model detected significant factor interactions. Post hoc pairwise comparisons were made between groups within time point and within groups between time points. Statistical software SAS^®^ Enterprise Guide^®^, Graph Pad Prism 5.01 and Sigmaplot 15 were used for the statistical analyses.

## Results

### General observations

The tumors grew fast with high variability causing many animals to be euthanized due to tumor size and as a result the study duration was 3.8 ± 1.2 weeks for the tumor-bearing animals. Neither DOX nor ET affected the animal survival (*P* = 0.85, *P* = 0.84, respectively, interaction *P* = 0.75). DOX decreased mouse running activity over time in the TDE-group compared to TE and CE groups (Figs. 1B and 2.2 ± 1.7, 3.7 ± 3.16, 4.2 ± 0.9 mean km/day ± SD, respectively).

Within tumor-bearing animals DOX decreased mouse body weight (Fig. 1C) and the relative heart weight to tibia length (RHW, Fig. 1D), wheel-running exercise had no effect on these variables. The same variables for the examination of the cancer effects alone and exercise effects without DOX are shown in Supplemental Fig. 1 Additional File 1. Within no-DOX groups, ET increased RHW (*P* < 0.001), which was most prominent in the CE-group, whereas subcutaneous mammary tumors decreased the RHW (*P* = 0.034) but did not affect animal body weights.

DOX decreased relative gastrocnemius weight to tibia length (RGW) but neither ET nor tumor-burden affected RGW (Supplemental Tables 3–4, Additional File 1). The ET effect was verified by measuring gastrocnemius CS activity (Supplemental Tables 3–4, Additional File 1). Additional gastrocnemius data is provided in the Supplemental Tables 3–4, Additional File 1.

### Echocardiogram results

**Fig. 2:**
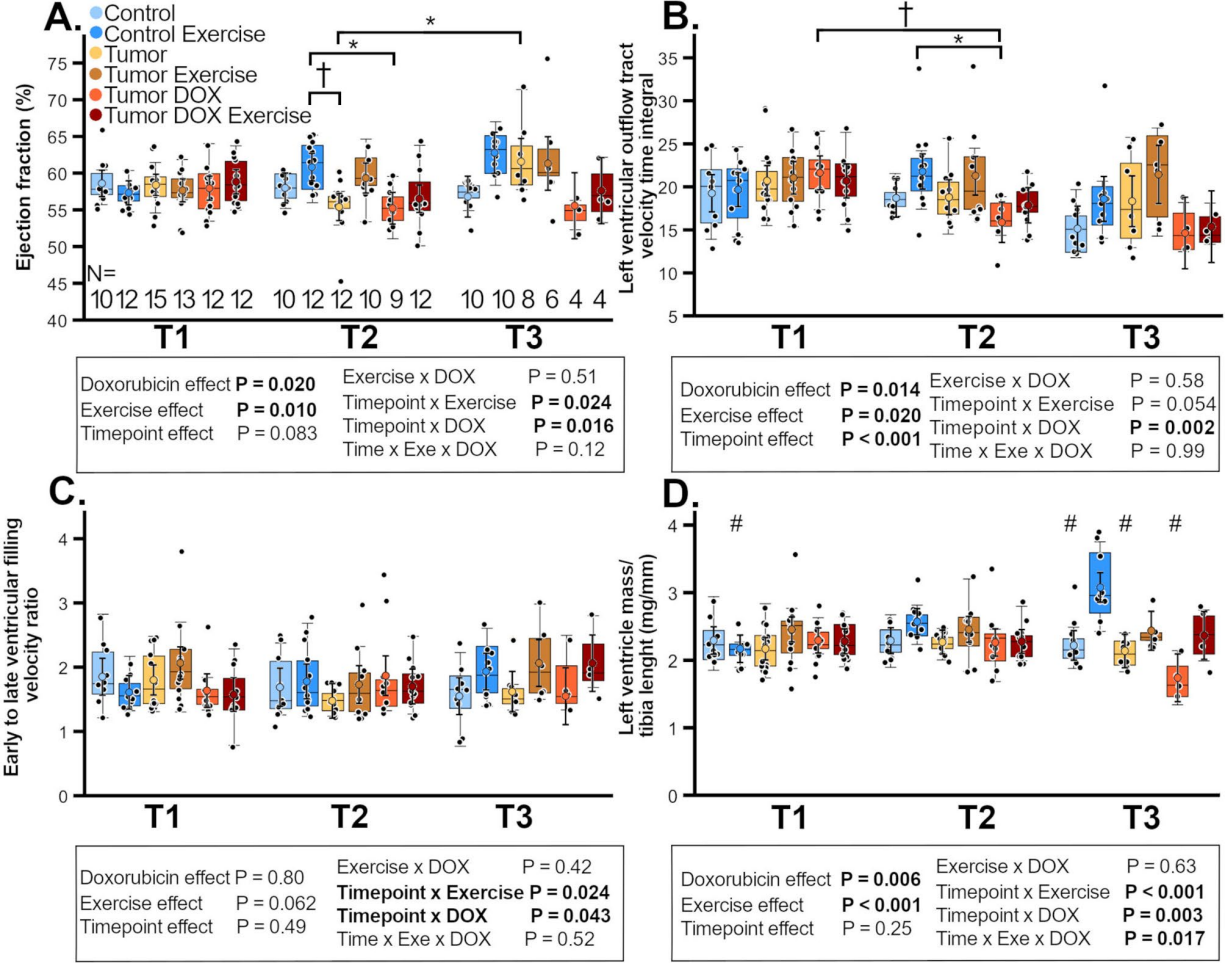
Changes in mouse cardiac function over time. Ejection fraction (**A**), outflow track velocity time integral (**B**), early to late filling velocity ratio (**C**) and left ventricle mass relative to tibia length (**D**) in female FVB-mice with or without subcutaneous I3TC-tumors or DOX treatment at baseline (T1), 2.8 weeks (T2) and 4.8 weeks (T3) after tumor inoculation. Panel A has group n-numbers (N) indicated with numbers on the x-axis below each group boxplot. Linear mixed model *P* < 0.05 are highlighted in bold with colored circles showing model-predicted values ± CI-95%. Holm-Sidak: *P < 0.05, †*P* < 0.01

When comparing all groups, DOX decreased time dependently the LV EF, LVOT VTI and relative LV-mass to tibia length (RLV-mass) (Fig. 2). Post hoc test revealed that at T2 EF was significantly lower in TD-group compared to CE-group, LVOT VTI was significantly lower in TD-group at T2 compared to T1 while RLV-mass of TD-group at T3 was significantly smaller compared to TE-group.

Meanwhile, ET increased LV EF and RLV-mass over time, and there was a tendency towards higher LVOT VTI in exercised animals (Fig. 2). The post hoc comparison revealed that EF of CE-group was significantly greater at T2 compared to T-group and TD-group while EF of TE-group was higher in T3 compared to T2. Notably TDE-group EF did not differ from the other groups, suggesting that exercise blunts the DOX-effect on EF. Similarly, post hoc found no statistical difference between baseline and following timepoints in TDE-group unlike within TD-group. Importantly, there was a three-way time×exercise×DOX interaction on RLV-mass with the post hoc analysis revealing ET increased RLV-mass prominently in CE-group, with the RLV-mass of CE-group being greater compared to all unexercised groups at T3, suggesting that ET can blunt the DOX-induced reduction of RLV-mass.

There was time×exercise and time×DOX interaction, with E/A increasing over time with ET and with DOX but without post hoc finding significant alterations between timepoints or groups (Fig. 1D). The comparison of no-DOX groups further confirmed that ET increased EF, LVOT-VTI and RLV-mass while there was no ET effect on E/A ratio (*P* = 0.056, Supplemental Fig. 2, Additional File 1). The comparisons of no-DOX groups also confirmed that ET increases EF particularly within CE-group. Post hoc test revealing that tumor-burden blunts ET-induced cardiac hypertrophy as the CE-group RLV-mass was significantly greater compared to TE-group at T3.

Importantly, within no-DOX groups tumor-burden had no effect on EF, LVOT VTI, E/A ratio, or RLV-mass (Supplemental Fig. 2A-F, Additional File 1). The heart rate is presented in Supplemental Fig. 3A-B, Additional File 1.

In summary, DOX decreased cardiac systolic function and LV-mass while exercise mitigated these effects with cancer having no effect aside blunting exercise-induced physiological LV hypertrophy.

### LVGU and enzyme activities

**Fig. 3:**
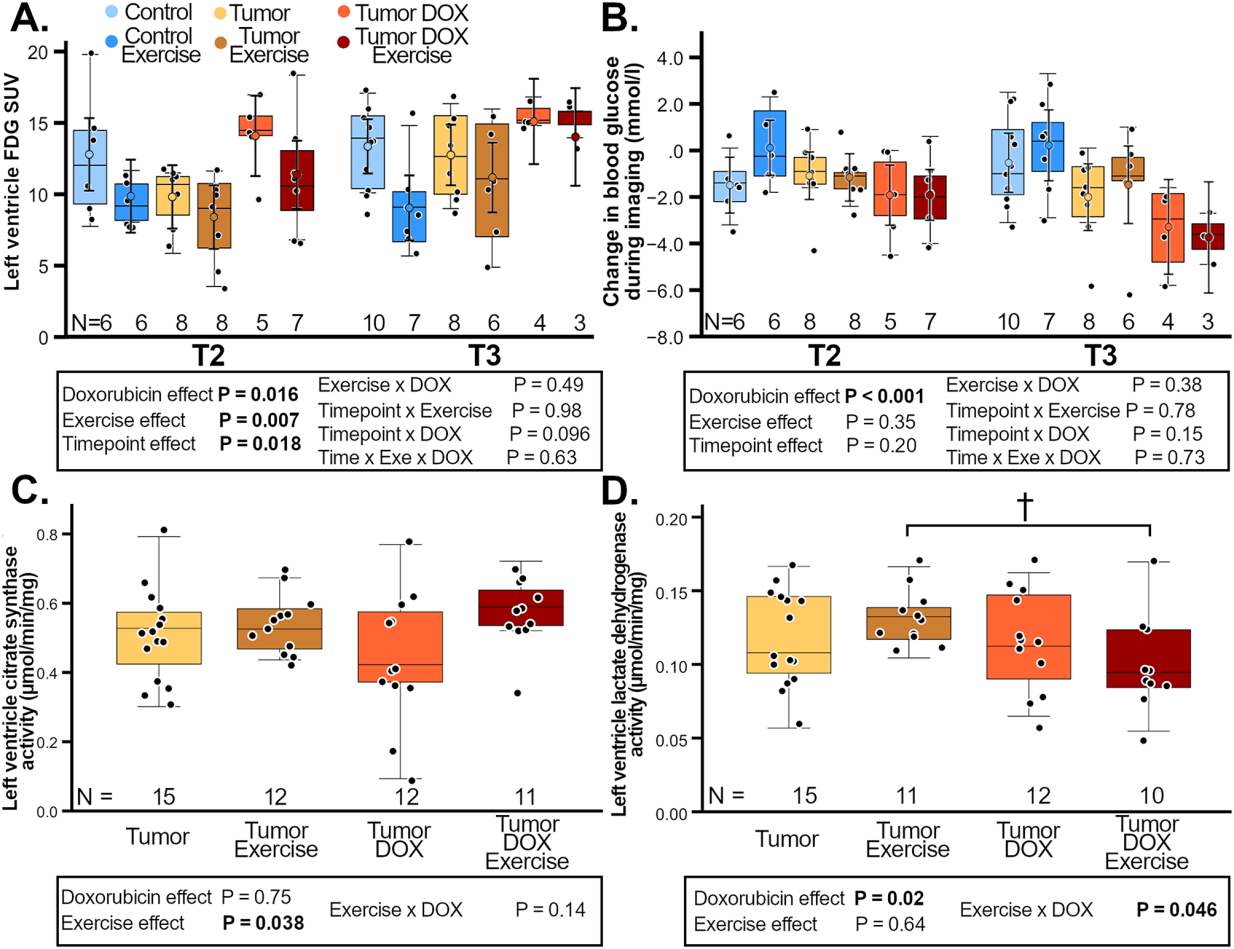
Cardiac glucose metabolism markers. Groups are female FVB-mice with or without I3TC-tumors or DOX treatment with group n-numbers (N) indicated on the x-axis below each group boxplot. Left ventricle FDG–SUV indicative of glucose uptake (**A**) and blood glucose change during PET-imaging (**B**) are shown 2.8 weeks (T2) and 4.8 weeks (T3) after tumor inoculation with enzyme function at euthanasia (**C**-**D**). Linear mixed model (A-B) or two-way ANOVA (C-D) *P* < 0.05 are highlighted in bold with colored circles showing model-predicted values ± CI-95% (A-B). Holm-Sidak: †*P* < 0.01

The average fasting blood glucose during the FDG-PET imaging did not differ between the groups over time (Supplemental Fig. 3C-D, Additional File 1). DOX increased LVGU over time and ET decreased it (Fig. 3A); suggesting that ET blunted some of the DOX-induced increase in LVGU.

Although average fasting blood glucose did not differ between groups during PET-imaging, the blood glucose decreased more in DOX-treated animals during PET-imaging compared to no-DOX tumor-bearing animals while ET had no effect (Fig. 3B). Within the tumor-bearing groups ET increased the LV CS activity in both exercising groups compared to both non-exercising groups (Fig. 3C). Meanwhile there was DOX×exercise interaction on LV LDH activity so that ET decreased LDH-activity only in DOX-treated mice (Fig. 3D).

In no-DOX groups ET decreased LVGU over time and did not affect change in blood glucose during PET-imaging, while ET increased LV LDH activity (Supplemental Fig. 4, Additional File 1). Interestingly, there was no significant ET effect on LV CS activity within no-DOX groups, suggesting more prominent ET effect in TDE-group. Tumor-burden did not affect LV CS activity, LDH activity, and LVGU. However, within no-DOX groups there was time×cancer interaction on LVGU, with LVGU increasing over time within both tumor-bearing groups together compared to both tumor-free groups, in which LVGU remained constant. Additionally, tumor-bearing groups showed a decrease in blood glucose during PET-imaging compared to both tumor-free groups.

**Fig. 4:**
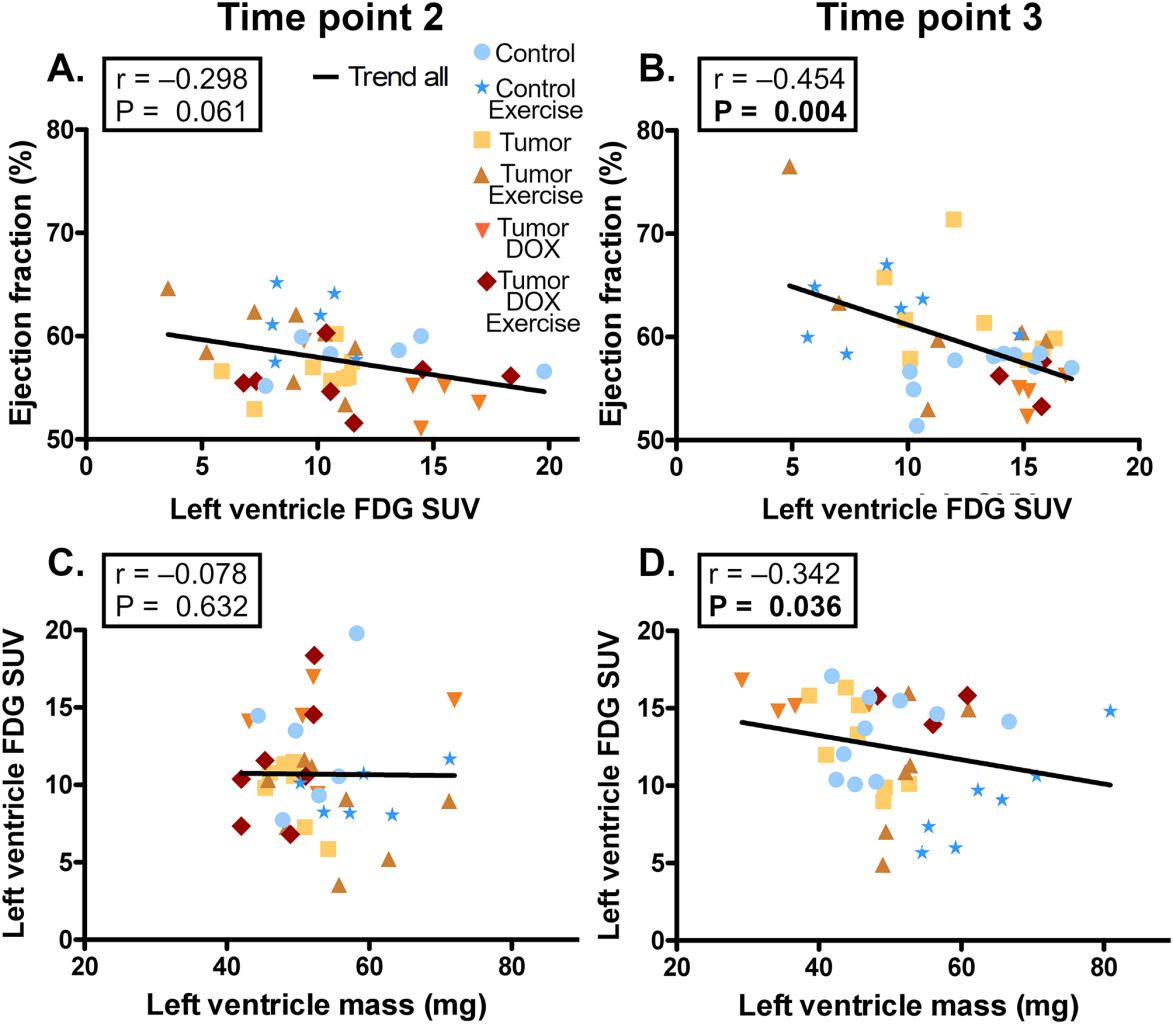
Left ventricle glucose uptake (FDG–SUV), function and mass. Correlations between variables 2.8 weeks and 4.8 weeks after tumor inoculation (time points 2–3) in female FVB-mice with or without subcutaneous I3TC-tumors or DOX treatment. Spearman-correlation *P* < 0.05 are highlighted in bold. N-numbers for control, control exercise, tumor, tumor exercise, tumor DOX and Tumor DOX exercise are 6, 6, 8, 8, 5 and 7 at T2 and 10, 7, 8, 6, 4 and 3 at T3 respectively

Additionally, there was a negative correlation between LV EF and the LVGU at T3 but this correlation was not quite significant earlier at T2 (Fig. 4A-B). Similarly, there was no significant correlation between the LVGU and the RLV-mass at T2, whereas within T3 the LVGU had a negative correlation with RLV-mass (Fig. 4C-D). Interestingly, the negative correlation between LVGU and RLV-mass at T3 was present in all groups except CE group, which had positive variable relationship. T3 LV EF also positively correlated with LV mass (Supplemental Fig. 5A, Additional File 1).

In summary, DOX and cancer increased LV glucose uptake decreasing blood glucose during PET-imaging with exercise decreasing LV glucose uptake.

### Mitochondrial function in the LV homogenate

DOX increased mitochondrial LEAK and decreased coupling efficiency and CS/CIV ratio in the tumor-bearing animals (Table 1). ET did not significantly affect these parameters, although there was a trend of LEAK decreasing and coupling efficiency increasing with ET. Furthermore, at T3 the LV EF negatively correlated with LEAK and positively with mitochondrial coupling (Supplemental Fig. 5B-C, Additional File 1). All other mitochondrial parameters in LV were unaffected by DOX or ET in tumor-bearing mice (Table 1). Tumor-burden or ET had no significant effect within no-DOX groups on any of the mitochondrial function parameters (Supplemental Table 5, Additional File 1).

**Table 1:** The left ventricle mitochondrial respiration parameters

	Tumor *N* = 10	Tumor Exercise *N* = 8	Tumor DOX *N* = 12	Tumor DOX Exercise *N* = 9	DOX effect	Exercise effect	DOX × Exercise
LEAK (pmol/(s×mDNA)) (pmol/(s×mg)) (pmol/(s×CS))	0.004 ± 0.003 14.1 ± 11.9 4.5 ± 3.2	0.003 ± 0.002 12.8 ± 6.7 4.0 ± 2.1	0.007 ± 0.004 25.40 ± 11.79 14.3 ± 10.9	0.005 ± 0.003 17.7 ± 12.5 5.7 ± 3.9	**0.024** **0.030** **0.023**	0.097 0.23 0.14	0.53 0.38 0.12
CI-OXPHOS (pmol/(s×mDNA)) (pmol/(s×mg)) (pmol/(s×CS))	0.041 ± 0.016 141.6 ± 43.6 47.9 ± 8.0	0.037 ± 0.012 165.3 ± 38.2 51.0 ± 12.0	0.044 ± 0.014 156.2 ± 28.8 95.0 ± 88.9	0.047 ± 0.010 173.3 ± 42.7 58.4 ± 13.5	0.14 0.37 0.11	0.85 0.11 0.31	0.49 0.79 0.23
CI&CII-OXPHOS (pmol/(s×mDNA)) (pmol/(s×mg)) (pmol/(s×CS))	0.14 ± 0.03 492.6 ± 96.3 180.0 ± 66.8	0.12 ± 0.04 518.4 ± 97.19 160.8 ± 35.3	0.15 ± 0.05 541.1 ± 83.9 320.0 ± 270.3	0.14 ± 0.03 537.1 ± 99.8 181.0 ± 31.9	0.098 0.28 0.12	0.30 0.72 0.13	0.73 0.63 0.25
ETS max (pmol/(s×mDNA)) (pmol/(s×mg)) (pmol/(s×CS))	0.14 ± 0.03 513.2 ± 101.0 186.9 ± 66.6	0.12 ± 0.05 534.4 ± 101.7 165.6 ± 35.8	0.17 ± 0.05 580.6 ± 83.0 342.5 ± 286.4	0.15 ± 0.03 564.6 ± 106.9 189.9 ± 31.5	0.06 0.13 0.10	0.22 0.94 0.11	0.81 0.56 0.23
CII-ETS (pmol/(s×mDNA)) (pmol/(s×mg)) (pmol/(s×CS))	0.11 ± 0.02 390.1 ± 88.1 144.0 ± 61.2	0.09 ± 0.04 398.9 ± 75.5 123.8 ± 28.1	0.12 ± 0.04 435.2 ± 60.6 256.5 ± 212.4	0.11 ± 0.02 415.4 ± 79.9 139.5 ± 22.2	0.08 0.22 0.12	0.18 0.82 0.10	0.88 0.56 0.24
CIV max (pmol/(s×mDNA)) (pmol/(s×mg)) (pmol/(s×CS))	0.39 ± 0.10 1393.3 ± 257.5 507.4 ± 169.1	0.38 ± 0.2 1601.2 ± 547.6 491.1 ± 153.2	0.45 ± 0.2 1526.3 ± 474.7 918.2 ± 862.0	0.46 ± 0.2 1725.5 ± 701.6 575.9 ± 220.3	0.23 0.49 0.14	0.89 0.27 0.28	0.42 1.00 0.33
Cyt C response (%)	11.7 ± 12.0	10.7 ± 7.4	9.1 ± 6.0	12.4 ± 10.7	0.88	0.70	0.48
Coupling efficiency	0.92 ± 0.06	0.93 ± 0.03	0.86 ± 0.06	0.91 ± 0.06	**0.045**	0.07	0.27
(CS-activity/CIV) ×10^− 3^	0.39 ± 0.11	0.39 ± 0.12	0.31 ± 0.15	0.38 ± 0.12	**0.049**	0.46	0.65
Mitochondrial index	1823.8 ± 301	2490.3 ± 1359	1855.5 ± 364	1889.8 ± 403	0.21	0.12	0.16

Groups consist of subcutaneous I3TC−tumor bearing female FVB−mice with or without DOX treatment. All the values shown are mean±SD normalized to mitochondrial index, tissue weight and citrate synthase activity respectively unless stated otherwise. Two−way ANOVA P−values are shown with P−values <0.05 are highlighted in bold. CI−OXPHOS = Complex 1 linked oxidative phosphorylation CI&CII−OXPHOS = Complex 1 & Complex 2 linked oxidative phosphorylation, CII−ETS = Complex 2 linked electron transfer capacity, CIV max = maximal activity of complex 4, CS = citrate synthase, Cyt C = cytochrome C, DOX = doxorubicin, ETS max = maximal electron transfer capacity, LEAK = respiration

### Oxidative stress and anti-oxidative enzymes in the LV

Within the tumor-bearing animals neither DOX nor ET affected LV oxidative stress, or the LV anti-oxidative enzymes measured (Table 2). Similarly, neither ET nor tumor-burden affected LV oxidative stress or anti-oxidative enzyme activity within no-DOX groups (Supplemental Table 6, Additional File 1).

**Table 2:** Left ventricle oxidative stress, anti-oxidative enzymes, and metabolic enzyme activities

	Tumor	Tumor Exercise	Tumor DOX	Tumor DOX Exercise	DOX effect	Exercise effect	DOX × Exercise
HOAD (μmol/min/mg)	0.08 ± 0.04	0.10 ± 0.04	0.13 ± 0.08	0.09 ± 0.06	0.39	0.46	0.08
SOD-activity (%)	68.8 ± 4.4	68.3 ± 3.5	67.8 ± 5.6	68.8 ± 5.1	0.88	0.86	0.58
CAT-activity (μmol/min/mg)	34.6 ± 5.4	38.2 ± 6.7	37.6 ± 5.2	38.2 ± 8.4	0.38	0.27	0.34
LPX-level (μM/mg)	36.4 ± 5.4	37.9 ± 5.6	36.0 ± 4.5	35.0 ± 4.6	0.25	0.83	0.39
CARB-level (μmol/mg)	11.5 ± 2.1	11.5 ± 2.2	12.4 ± 2.6	11.0 ± 2.8	0.82	0.33	0.32
N-Number	15, 14^HOAD^	12, 11^SOD^	12, 11^CAT^	11, 12^SOD, LPX^			

Groups consist of subcutaneous I3TC-tumor bearing female FVB-mice with or without DOX treatment. All the values mean±SD are presented relative to protein amount unless stated otherwise with *P*-values for the two-way ANOVA tests. N-numbers apply to all assays unless stated otherwise with superscript. CARB = protein carbonylation, CAT = catalase, DOX = doxorubicin, HOAD = 3-hydroxyacyl-CoA dehydrogenase, LPX = lipid peroxidation indicated by lipid hydroxyl peroxides, SOD = superoxide dismutase

### LV histology

**Fig. 5:**
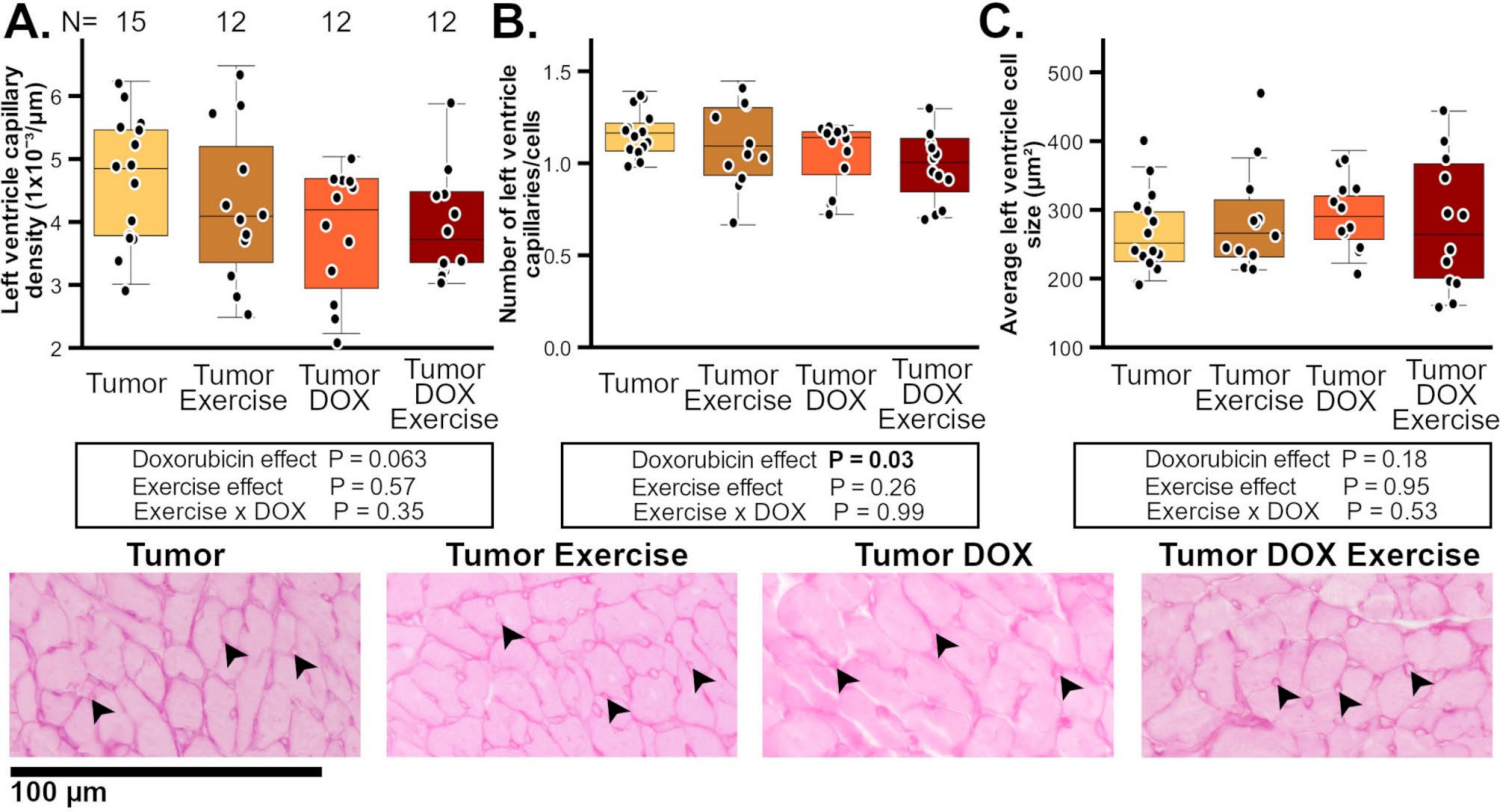
Left ventricle histology of I3TC-tumor-bearing female FVB-mice. Capillary density (**A**), capillaries per cell (**B**) and transversal cell size (**C**) of groups with or without DOX treatment. Exemplary PAS-stained histological 20× images with contrasts and tone adjusted for visibility only. Arrowheads denote capillary vessels and two-way ANOVA *P* < 0.05 are highlighted in bold. Panel A has group n-numbers (N) indicated with numbers above each group boxplot. The data obtained for current manuscript in A-C from the tumor group without exercise and without doxorubicin treatment has been previously published in Koivula et al. (2024) to support clinical findings [36]

DOX did not affect LV capillary density within the tumor-bearing animals, although there was a non-significant trend towards decreasing capillary density (*P* = 0.063, Fig. 5A). However, DOX did significantly decrease LV capillaries per myocardial cells in both DOX-treated groups compared to untreated groups (Fig. 5B). Within tumor-bearing mice neither DOX nor ET affected the average transversal LV cardiomyocyte size, nor did ET affect the LV capillary density or LV capillaries per cell (Fig. 5). Within no-DOX groups ET and tumor-burden had no effect on LV capillary density, number of capillaries per cell or cell size (Supplemental Fig. 6, Additional File 1).

## Discussion

The current study demonstrated that in DOX-treated tumor-bearing mice, short-term ET was associated with higher LV EF, greater LV-mass, lower LVGU, and lower LV LDH activity and higher CS activity, compared with non-exercised tumor-bearing DOX-treated mice. The increased LVGU indicated by increased FDG-uptake significantly correlates with loss of LV-mass and the decrease in EF. The lower LDH activity suggests decreased glycolysis or decreased lactate utilization and may contribute to the blunted increase in cardiac glucose uptake in DOX-treated exercised animals.

### Effects of DOX on cardiac and skeletal muscle

Expectedly, we found DOX causing loss of body weight, cardiac and skeletal muscle mass and to impair cardiac function and affect cardiac vasculature, which are all in agreement with previous findings [2–4, 20]. In the current study the reduced LV-mass and the impaired mitochondrial function likely contributed to the impaired cardiac function, which in turn together with the reduced gastrocnemius muscle mass contributed to the decreased physical activity of DOX-treated mice. Indeed, in our study LV EF positively correlated with the LVM and mitochondrial coupling and negatively with mitochondrial LEAK. DOX damages skeletal muscle contributing to skeletal muscle weakness thus affecting voluntary physical activity of mice [21]. DOX-treated breast cancer patients have reduced exercise behaviors [22], but there are contrasting results about DOX effects on rodent physical activity [23, 24]. The differential DOX-effect on physical activity could be due to differences in DOX-dosing and tumor burden. In the current study voluntary exercise was used, which is likely more comfortable than forced running, and thus more feasible for individuals with cancer. Furthermore, it has been shown that even short-term low-intensity ET is protective against DOX-induced toxicity in non-tumor-bearing mice [25, 26].

DOX has been shown to increase cardiac FDG-uptake as a measure for LVGU in both preclinical and clinical studies [11, 12]. In accordance with previous studies, we found DOX to increase cardiac FDG-uptake which is thus indicative of increased glucose uptake. This was supported by the greater decrease in blood glucose during FDG-PET imaging in the DOX-treated mice. Link between DOX-induced increase in LVGU and increased cardiac glucose consumption has been hard to confirm due to its possibly short duration compared to persistently increased FDG-uptake [12]. One in vitro study showing that DOX increases specifically FDG-uptake [13] while another in vitro study showed that for a short duration glucose consumption was also increased along with FDG-uptake [27]. In line with increased glucose consumption DOX has been shown to increase cardiac glycolytic activity [28]. We did not measure cardiac immune cell infiltration, but recruitment of inflammatory cells and their glucose utilization during tissue damage could increase FDG-uptake, although in one previous study DOX increased FDG-uptake also without increased inflammatory infiltrate [13].

In the current study DOX-treated mice showed increased mitochondrial electron leak, decreased coupling efficiency, decreased CS/CIV ratio in LV-homogenate, and decreased LV capillaries per myocardial cells, which can impair energy production and availability, thus making increased LVGU a possible compensatory mechanism to maintain cardiac energy metabolism. However, the LV EF inversely correlated with the cardiac LVGU, suggesting that increased glucose uptake does not serve to maintain cardiac function, and that the increased FDG-uptake works as a marker of cardiac dysfunction. This is further supported by previous studies which linked DOX-induced LVGU directly with the DOX-induced oxidative stress [12, 13]. Furthermore, the inverse correlation between EF and cardiac LVGU is supported by studies in cancer patients receiving anthracycline chemotherapy, who exhibited increased cardiac GU associated with decline in cardiac function [29, 30]. Our findings also showed a negative correlation between LVGU and LV-mass. This inverse relationship was not present in CE-group which could be explained by the prominent ET-induced LV-hypertrophy in this group.

Although previous evidence showed DOX to increase cardiac oxidative damage [4], we did not find increased oxidative damage of cardiac lipids or proteins. Some studies similarly reported no increase in cardiac lipid peroxidation or protein carbonylation in response to treatment with DOX [26, 31], which could be due to the timing of sample collection since DOX-administration, which in our study unfortunately varied between tumor-bearing animals due to tumor growth. DOX-induced oxidative damage is an early event [27] and might therefore be overlooked if measured after the oxidative damage has been repaired or when damaged cells have been removed via apoptosis.

### Effects of ET on heart and skeletal muscle

In the current study DOX decreased mouse running activity; however, there was an ET effect evidenced by the significantly increased gastrocnemius CS activity. FVB/NJ-mice have been shown to perform on average 4 km/night voluntary wheel-running [32], and in our study no-DOX mice ran similar distances. Although ET did not alleviate gastrocnemius atrophy in our study, the increased CS activity is a beneficial alteration for the skeletal muscle function.

Evidence supports that more intense ET, usually performed via forced treadmill exercise, and ET preconditioning can yield greater beneficial effects on the heart function, although lower intensity, i.e. voluntary wheel-running, was also beneficial when performed concomitantly with DOX treatment [3]. In one study, 6-week wheel-running attenuated the shift in myocardial contractile protein expression in DOX-treated non-tumor-bearing rats, despite their running activity declining approximately 50% by the end of the study [23]. Importantly, we found that nearly 4-week exercise blunted DOX-induced cardiac functional impairment and atrophy, despite running activity of mice declining by the end of the study. A meta-analysis of preclinical exercise intervention studies with varying levels of exercise supported the mitigating effects of exercise on DOX-induced cardiotoxicity [3]. However, the lowest exercise intensity among these studies (2-week treadmill running 450 m/day five days a week) did not mitigate DOX-induced cardiotoxicity in melanoma-bearing male mice [33]. The benefits of exercise may differ among the studies due to differences in DOX-dosing and the cancer model since cancer may alter the metabolic state with a wide array of tumor-secreted factors. For example, our study shows that despite CE-mice seemingly running less towards the end of the study compared to TE-mice, they have more LV-hypertrophy, suggesting that cancer may blunt ET-induced LV-hypertrophy, which could affect how well exercise can counter functional impairment of the myocardium. Tumor-burden alone also increased LVGU over time, which could be indicative of increased cardiac inflammation, further highlighting the importance of employing tumor models in DOX-cardiotoxicity studies. In line with this, blood glucose decreased more in tumor-bearing mice compared to tumor-free during PET-imaging, which is reasonable also considering tumor glucose uptake.

In the current study the seemingly higher running activity of the tumor-bearing no-DOX mice compared to tumor-free mice is likely due to greater running underestimation in tumor-free mice towards the end of the study. At the beginning of the study the running distances were underestimated similarly for all mice because mice were housed in pairs to improve mouse welfare, and the running-activity was assumed equal and halved for calculation activity per mouse although mice could use wheel simultaneously. Due to tumor growth some mice were euthanized earlier, leaving some tumor-bearing animals housed alone causing less underestimation of their running activity.

Previously we found that greater mouse running activity with shorter ET duration can increase the LV capillarity of DOX-treated animals with lower cumulative DOX-dose [34]. Previously we also found lower wheel-running activity at a shorter duration to increase cardiac capillarity in melanoma-bearing male mice [35]. However, in the current study ET did not improve LV capillarity even within the no-DOX groups with higher physical activity. These differing results could be explained by variable DOX-dosing, exercise intensity and high variability within tumor-bearing groups.

To our knowledge, our study is the first to report that exercise can blunt the DOX-induced increase in LVGU. ET did not however reduce the DOX-induced mitochondrial uncoupling, although there was a trend towards decreased LEAK and increased coupling efficiency in all tumor-bearing exercised mice. Our results indicate that in the exercised tumor-bearing DOX-treated animals, the cardiac muscle shifts to use preferentially glucose via aerobic pathway as was evidenced by the increased cardiac CS activity and reduced cardiac LDH activity, signifying possibly reduced glycolysis. However, we measured overall LDH activity without differentiating its subtypes, which means that decreased LDH activity in exercised DOX-treated animals could also signify reduced cardiac usage of lactate. Previously DOX has been shown to increase cardiac glycolysis [28], which could contribute to DOX-mediated increase in LVGU. However, in our current study the LDH activity was not increased in unexercised DOX-treated mice, which could be because of different running duration/activity and DOX-dosing. Differing from our current results, our previous study showed increased LV LDH activity in DOX-treated mice with higher wheel-running activity in shorter duration with smaller cumulative DOX-dose [34]. Similarly, in the current study LV LDH activity increased in exercised no-DOX mice which also ran more than DOX-treated mice. Differing exercise intensity and duration could explain the differences in our previous findings and/or that the changes in total LV LDH activity reflect the increase in the activity of different LDH subtypes. Further investigation is needed into the contribution of different LDH-subtypes in relation to cardiac glucose uptake in DOX-mediated cardiotoxicity. More studies are also needed on what causes the increased mitochondrial uncoupling (increased LEAK), for example it could be speculated that there might be changes in uncoupling protein expression. It is possible that increased LEAK could drive increased glycolysis since LEAK makes the electron transfer chain less efficient at producing energy aerobically.

### Limitations

Firstly, the mouse running activities might be underestimated because the mice were housed in pairs (to reduce stress) and assumed to run equally thus halving the wheel readings, although mice were witnessed using the wheel also simultaneously. Voluntary ET is also a compromise as the exercise amount cannot be controlled; however, it allows mice to move at a comfortable intensity according to their natural circadian rhythm, thus reducing stress. Secondly, the use of non-orthotopic breast cancer model is a limitation, as the results might be different from the orthotopic primary tumor model, which may have different profile of tumor-secreted factors. Non-orthotopic model was used to avoid tumor location affecting mouse running activity and inducing additional stress. However, subcutaneous models account for some tumor-secreted factors and mimic a tumor which has spread from mammary tissue to nearby regions. Thirdly, the different euthanasia times of the tumor-bearing mice may increase the variability of endpoint parameters, making it harder to detect smaller differences between the groups. The animals were euthanized based on tumor size, thus there was no direct selection according to cardiac responses, which were the focus of the current study. Different euthanasia times could introduce survivor bias at T3-measurements, representing animals with the slowest tumor growth or with the best treatment/exercise response.

## Conclusions

In DOX-treated tumor-bearing mice, short-term voluntary wheel-running ET was associated with lower cardiac GU, higher EF, greater LV-mass, higher CS activity and lower LV LDH activity compared with non-exercised tumor-bearing DOX-treated mice. Further studies are needed to translate these findings into clinical level, and to determine whether ET-induced reduction of LVGU could predict improvement of cardiac outcomes in DOX-treated patients. Future preclinical studies should more often employ tumor-bearing models.

## Electronic supplementary material

Below is the link to the electronic supplementary material.

Supplementary Material 1: Additional file 1 consists of detailed materials and methods as well as additional supportive data in figures and tables. The additional figures and tables provide data and statistical comparisons for testing the effects of cancer alone which support and supplement the findings of the manuscript on the effects of doxorubicin in the tumor bearing animals. The additional figures and tables also contain data that was used to verify the training effect on the animals as well as additional skeletal muscle data. Supplemental data verifying cardiac functional changes association with changes in cardiac mass and mitochondrial function are also provided

## Data Availability

The datasets used and/or analyzed during the current study are available from the corresponding author on reasonable request.

## Abbreviations

CS: Citrate synthase
DOX: Doxorubicin
E/A: Early to late ventricular filling velocity ratio
ET: Exercise training
FDG: 2-[^18^F]fluoro-2-deoxy-D-glucose
LDH: Lactate dehydrogenase
LVGU: Left ventricle glucose uptake
LVOT VTI: Left ventricular outflow tract velocity-time integral over time
PET: Positron emission tomography
