# Correction to: Exercise training partly ameliorates cardiac dysfunction in mice during doxorubicin treatment of breast cancer

**DOI:** 10.1186/s12967-026-08273-0

**Published:** 2026-06-01

**Authors:** Tytti-Maria Uurasmaa, Pauline Bourdin, Wail Nammas, Shiva Latifi, Heidi Liljenbäck, Antti Saraste, Olli Eskola, Johan Rajander, Anne Roivainen, Helene Rundqvist, Anu Autio, Ilkka Heinonen, Katja Anttila

**Affiliations:** 1 Department of Biology, University of Turku, Turku, Finland; 2 Turku PET Centre, University of Turku, Turku University Hospital, Turku, Finland; 3 Turku Center for Disease Modeling, University of Turku, Turku, Finland; 4 Heart Centre, Turku University Hospital and University of Turku, Turku, Finland; 5 Accelerator Laboratory, Åbo Akademi University, Turku, Finland; 6 InFLAMES Research Flagship, University of Turku, Turku, Finland; 7 Department of Laboratory Medicine, Karolinska Institute, Stockholm, Sweden

**Correction to: Journal of translational medicine (2025) 23:89**

**https://doi.org/10.1186/s12967-025-06108-y**

In the sentence beginning “Results DOX increased LV glucose …” in this article, the [Results DOX increased LV glucose …] should have read

“Results DOX increased LV glucose uptake (LVGU) and mitochondrial uncoupling and decreased running activity, LV-weight, and ejection fraction (EF). In DOX-treated group ET blunted the increase in LVGU, increased LV-weight and EF, and lowered LV lactate dehydrogenase activity. Exercised mice had lower LVGU compared to unexercised groups and DOX-treated ET-group did not differ from tumor-free ET-group in LV-weight or EF whereas unexercised DOX-treated group did. ET also increased LV citrate synthase activity in tumor-bearing animals. There was an inverse association between LVGU and EF and LV-weight.”

In the sentence beginning “Additionally, there was a negative correlation …” in this article, the [Additionally, there was a negative correlation …] should have read

“Additionally, there was a negative correlation between LV EF and the LVGU at T3 but this correlation was not quite significant earlier at T2 (Fig. 4A-B). Similarly there was no significant correlation between the LVGU and the RLV-mass at T2, whereas within T3 the LVGU had a negative correlation with RLV-mass (Fig. 4C-D). Interestingly, the negative correlation between LVGU and RLV-mass at T3 was present in all groups except CE group, which had positive variable relationship. T3 LV EF also positively correlated with LV mass”. (Supplemental Fig. 5A, Additional File 1).

In this article, Figs. 3 and 4 appeared incorrectly and have now been corrected in the original publication. For completeness and transparency, the old incorrect version is displayed below.

Incorrect Figs. 3 and 4

**Figure 1:**
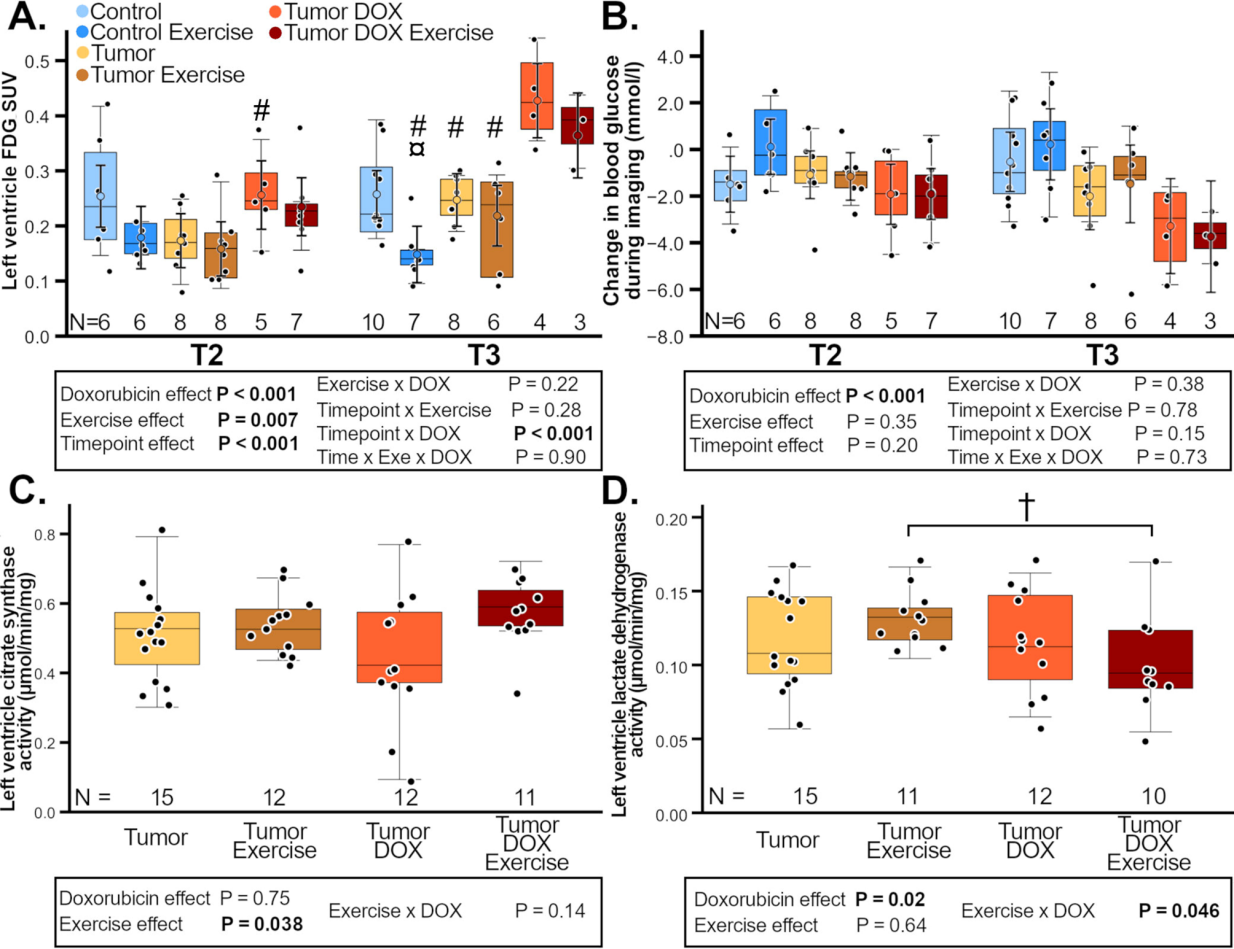

**Figure 2:**
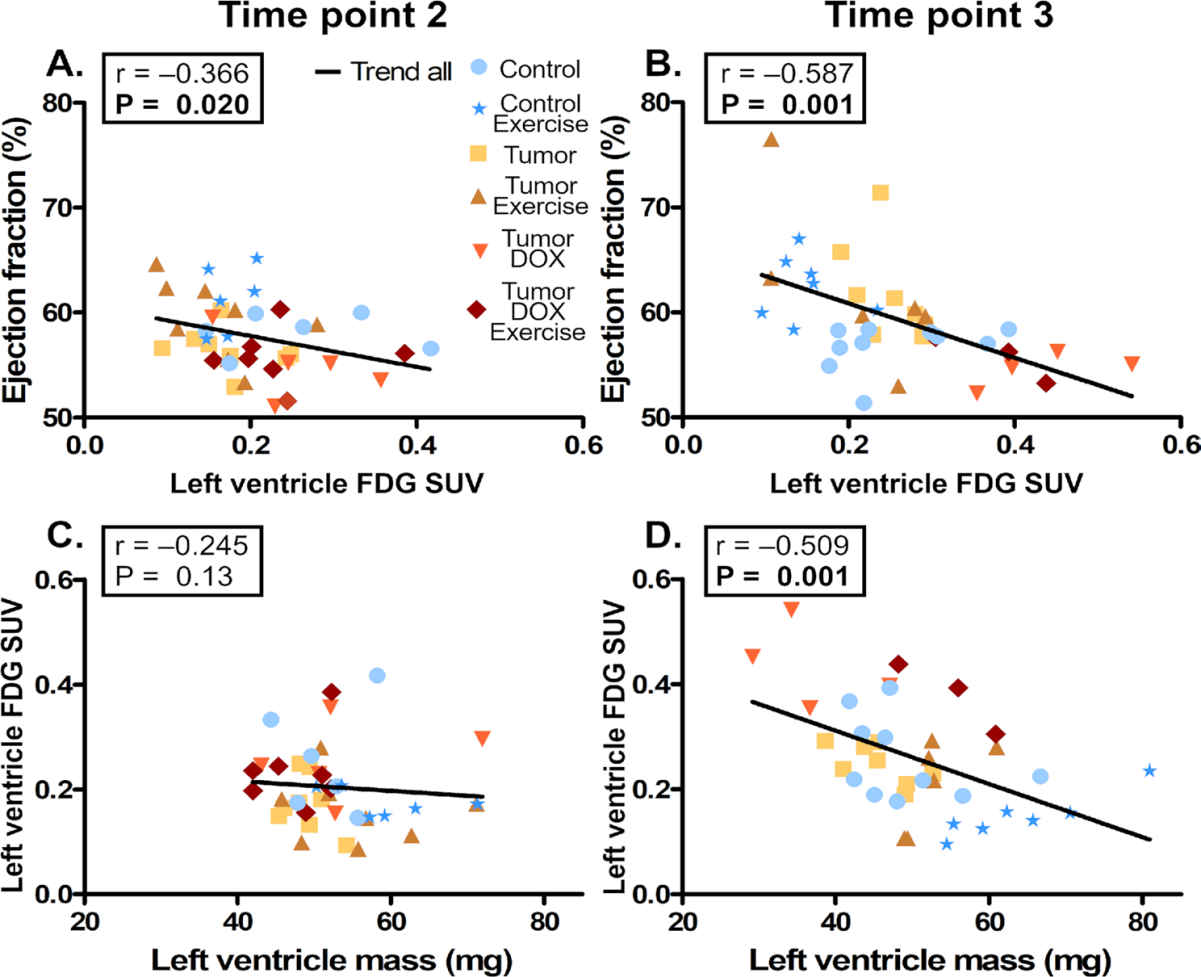

Correct Figs. 3 and 4

**Figure 3:**
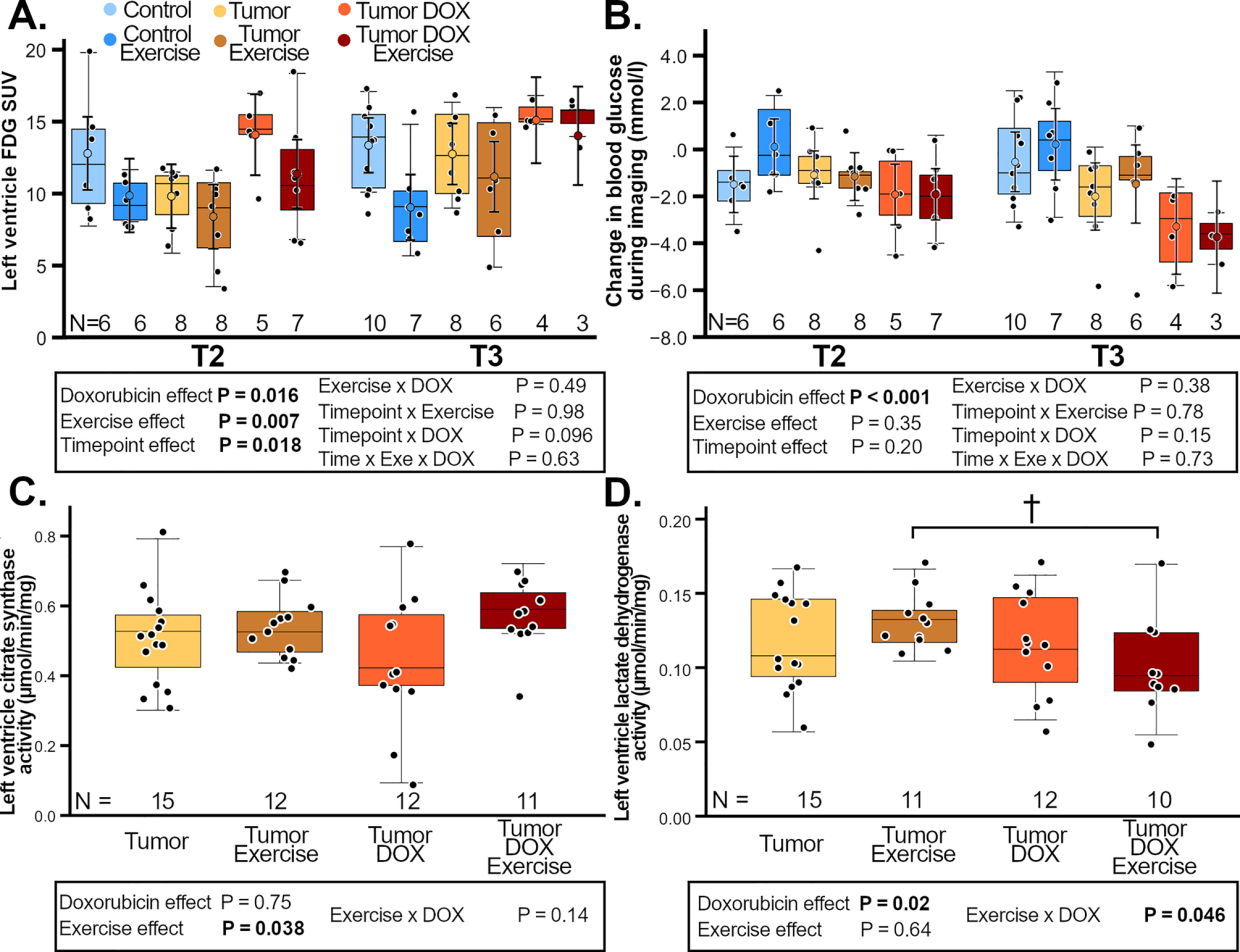

**Figure 4:**
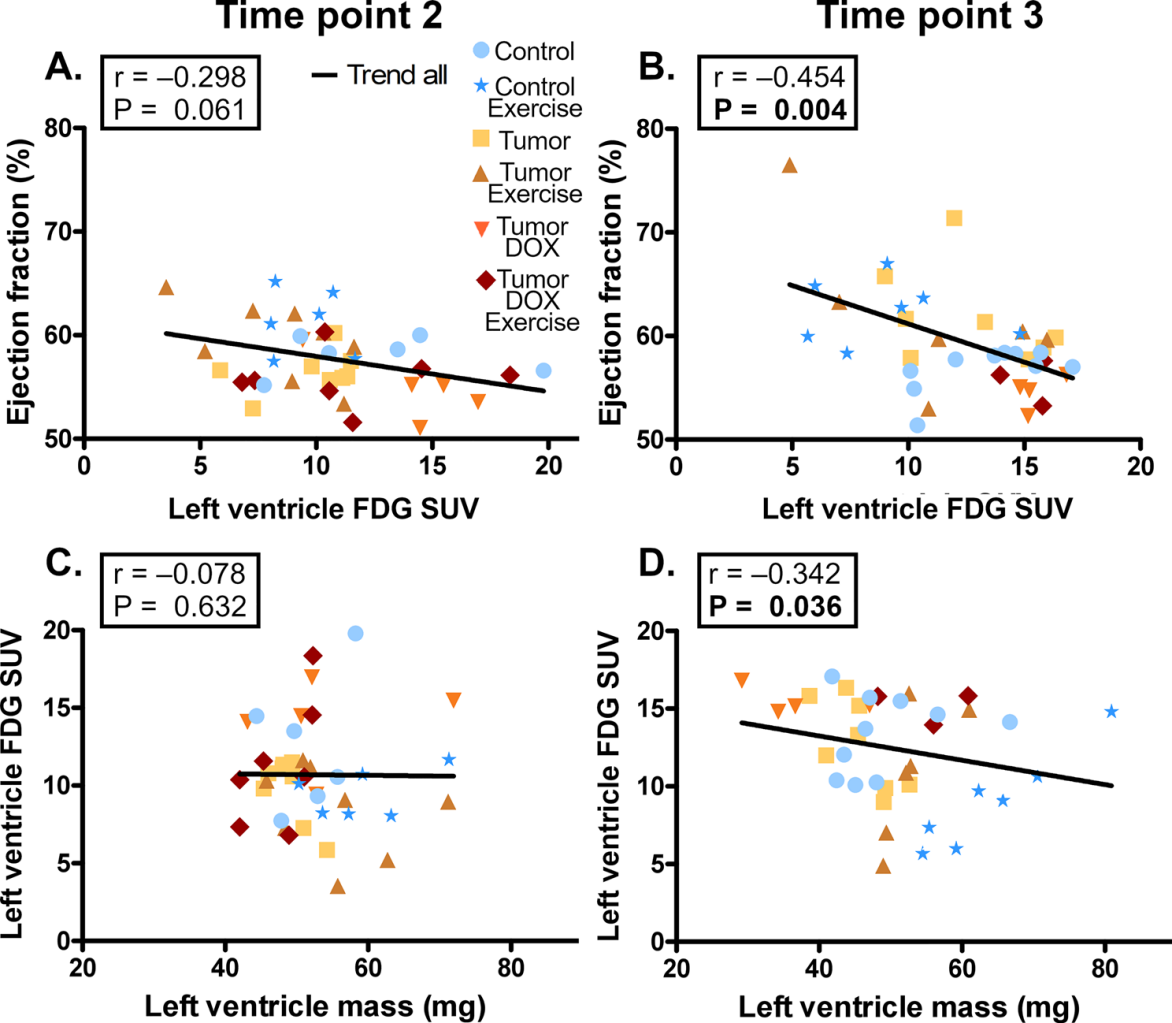

The incorrect figure in the Supplementary file was originally published with this article; it has now been replaced with the correct figure.

## Electronic supplementary material

Below is the link to the electronic supplementary material.

Supplementary Material 1

