# Supplementary material for: Exercise training partly ameliorates cardiac dysfunction in mice during doxorubicin treatment of breast cancer
Source: J Transl Med. 2025 Jan 21;23:89. doi: 10.1186/s12967-025-06108-y (PMC11748283; doi:10.1186/s12967-025-06108-y)
Supplement: Supplementary file 1 — Supplementary Material 1: Additional file 1 consists of detailed materials and methods as well as additional supportive data in figures and tables. The additional figures and tables provide data and statistical comparisons for testing the effects of cancer alone which support and supplement the findings of the manuscript on the effects of doxorubicin in the tumor bearing animals. The additional figures and tables also contain data that was used to verify the training effect on the animals as well as additional skeletal muscle data. Supplemental data verifying cardiac functional changes association with changes in cardiac mass and mitochondrial function are also provided [file 12967_2025_6108_MOESM1_ESM.pdf]

Additional file 1

Exercise training partly ameliorates cardiac dysfunction in mice during doxorubicin treatment of breast cancer

Tytti-Maria Uurasmaa[a,b*], Pauline Bourdin[a], Wail Nammas[b,d], Shiva Latifi[a,b], Heidi Liljenbäck[b,c], Antti Saraste[b,d], Olli Eskola[b], Johan Rajander[e], Anne Roivainen[b,c,f], Helene Rundqvist[g], Anu Autio[b], Ilkka Heinonen[b#], Katja Anttila[a#]

a. Department of Biology, University of Turku, Turku, Finland
b. Turku PET Centre, University of Turku and Turku University Hospital, Turku, Finland
c. Turku Center for Disease Modeling, University of Turku, Turku, Finland
d. Heart Centre, Turku University Hospital and University of Turku, Turku, Finland
e. Accelerator Laboratory, Åbo Akademi University, Turku, Finland
f. InFLAMES Research Flagship, University of Turku, Turku, Finland
g. Department of Laboratory Medicine, Karolinska Institute, Stockholm, Sweden

*Corresponding author
# Shared last authorship

Detailed materials and methods

Animals and the experimental protocol

The animal studies were approved by the national Project Authorization Board (permission number ESAVI/26508/2021) and were carried out in compliance with the EU Directive 2010/EU/63 on the protection of animals used for scientific purposes. Ten–week old female FVB/NHan®Hsd (Inotiv, Venray, Netherlands) mice (n=74, 21±1.5 g) were randomly divided into six groups: tumor-free control (C, n=10); tumor-free control with exercise (CE, n=12); I3TC-tumor group (T, n=15); I3TC-tumor group with exercise (TE, n=13); I3TC-tumor group with doxorubicin (DOX) treatment (TD, n=12); I3TC-tumor group with DOX treatment and exercise (TDE, n=12). Power calculation was not done as this was an explorative study, the sample size was chosen according to review of mouse studies on exercise effects on DOX induced cardiotoxicity, majority of which had sample size of 7-13 animals[1].

The experimental protocol (Figure 1A) was as follows: one day following the cancer cell inoculation all the animals gained full access to a low-profile wireless running-wheel (1/cage, Med Associates Inc ENV-044, St Albans, USA) with a stopper added to no-exercise groups' wheels to prevent spinning. The running activity was recorded using Med Associates Inc software (SOF-860, SOF-861). The animals were housed in standard conditions (2/cage) with *ad libitum* access to standard chow and tap water with 12-hour light-dark cycle. The tumor size was measured in 3 dimensions (length*width*depth) twice a week under isoflurane anesthesia (2%) using ultrasound (Vevo 2100, VisualSonics, Inc., Toronto, ON, Canada). A week after the cancer cell inoculation, the DOX (Doxorubicin accord 2 mg/ml, Vnr 510687) treatment was started with a dose of 5 mg/kg once a week intraperitoneally, while the test groups without chemotherapy received the same volume of phosphate-buffered saline (PBS). Throughout the study all animals were handled similarly. The animals were euthanized by cervical dislocation and organs were collected one week after the fourth DOX-dose or earlier if the tumor diameter reached 1.5 cm. Only one animal had to be euthanized early due to ulceration of the tumor. Tissues were rapidly collected from all the mice after euthanasia,

but due to technical difficulties the tissue samples were not collected from three of the mice. The running activity was calculated with the assumption that both mice would run equally in the cage, which may underestimate the running activity as the mice have been seen using the wheel simultaneously.

Cell culture and cell inoculation
Prior to inoculation, the I3TC cells were cultured for 7 days at +37˚C in 5% CO_2 in Dulbecco's Modified Eagle Medium (Thermo Fisher Scientific, 4.5 g/l D-Glucose, L-Glutamine REF41965-039) with 50 U/ml penicillin/streptomycin (EuroClone ECB3001D) and 10% fetal bovine serum (Sigma-Aldrich F7524-500 ml). On passage 4, the cells were detached and suspended in PBS for inoculation. The mice were anesthetized with isoflurane (2%) and subcutaneously inoculated with 1.8×10^6 I3TC tumor cells, originating from the PyMT-MMTV transgenic tumor model and isolated in a previous study by Weiland et al. (2012)[2]. The cells were obtained from the same batch as the ones used by Rundqvist et al. (2020)[3]. The control animals were injected with same volume (100 µl) of PBS under isoflurane anesthesia.

Echocardiography
All mice underwent transthoracic echocardiography at baseline (T1), 2.8 weeks after tumor cell inoculation (T2) and 4.8 weeks after tumor cell inoculation (T3, Fig 1A). Echocardiography was performed using a dedicated small animal Doppler ultrasound machine (Vevo 2100, VisualSonics, Inc., Toronto, ON, Canada) with a linear 13-24 MHz (MS250) transducer. Mice were examined on a 37˚C heated base, under light isoflurane inhalation anesthesia, with gas flow adjusted between 1.6-1.7 l/min without inducing bradypnea or bradycardia. The left ventricular end-diastolic and end-systolic volumes were measured in parasternal long-axis view by tracing the endocardial border of the left ventricle at end-diastole and end-systole, respectively. The 2-dimentional ejection fraction was calculated. The left ventricular end-diastolic and end-systolic diameters and the interventricular septal and posterior wall thickness were measured in M-mode parasternal long-axis view. M-mode ejection fraction and left ventricular mass were calculated. The aortic and mitral valve flows were assessed by continuous wave Doppler tracing with the Doppler beam aligned to the direction of flow, guided by color flow mapping parasternal long-axis view. Peak aortic flow velocity, as well as mitral early diastolic (E) and late diastolic (A) blood velocity were measured to calculate the left ventricular output tract velocity time integral (LVOT VTI) and E/A ratio.

PET/CT-imaging
Randomly chosen subset of mice was imaged with FDG PET/CT at two time points, 2.8 weeks after tumor cell inoculation (T2) and 4.8 weeks after tumor cell inoculation (T3). In the case that mouse was euthanized prior to the final time point, another random mouse from the same group was picked for last imaging. The mice were fasted for approximately 2 hours before the PET/CT-imaging. Mice were imaged two at a time, anesthetized using isoflurane (4-5 % induction, 1.5–2.5 % maintenance) and injected with 2-[^{18}F]fluoro-2-deoxy-*D*-glucose (FDG, 3.0±0.2 MBq; 140±15 MBq/kg i.v.) via tail vein cannula and then positioned to the heated bed of the small animal PET/CT (Molecubes NV, Ghent, Belgium). Mice were CT-scanned for anatomical reference and for attenuation correction. Static PET-imaging took place 20 minutes after the tracer injection with framing 1×1200s. Blood glucose levels were measured before and after the PET-imaging with a glucometer, (Bayer Contour XT, Bayer AG, Leverkusen, Germany). PET-data were reconstructed using iterative three-dimensional ordered subsets expectation maximization algorithm with automatic co-registration of PET and CT -images and confirmed visually based on anatomical landmarks. The PET/CT images were analyzed with Carimas-software[4] (Turku PET Center, Turku, Finland). The regions of interest (ROIs) were defined manually for the left ventricle on the PET/CT-images, using the CT as an anatomical reference. The left ventricle (LV) standardized FDG-uptake values (FDG-SUVs)

normalized for the injected radioactivity dose and animal body weight as a measure for LV glucose uptake were calculated using Carimas-software.

Mitochondrial respiration from tissue homogenate

The mitochondrial oxygen consumption rate measurement protocol from tissue homogenates was modified from Cantó & Garcia-Roves (2015)[5]. Immediately after euthanasia by cervical dislocation the mouse heart was placed on ice-cold biopsy preservation solution (BIOPS, Supplemental Table 1). The apex of the heart was cut for mitochondrial measurement and sliced into small pieces with the epicardium carefully removed. Tissue pieces were blotted dry, weighed, and transferred into cold mitochondrial respiration medium (Mir05, Supplemental Table 1) and rapidly shred to pieces (2×30 s) using scissors, with 2-minute incubation on ice between the shredding bouts. The shredded tissue was further homogenized by completing two downwards strokes at speed one with homogenizing pestle attached to Heidolph Elektro KG stirrer (Kelheim, Germany). The homogenate was diluted to desired concentration and 0.5 mg of tissue was transferred to the high-resolution respirometry oxygraph 2k (Oroboros Instruments corp., Innsbruck, Austria) measurement chamber. Catalase 280 U/ml (Sigma-Aldrich C40) was added to each chamber and the chambers were oxygenated to 300 µM O_2-level by injecting pure oxygen to the airspace of the half-closed chamber. The substrates and inhibitors were added sequentially according to Supplemental Table 2 and the oxygen flux was allowed to stabilize before addition of the next substrate or inhibitor. The oxygen fluxes corresponding to uncoupled proton leak, complex 1-2 linked oxidative phosphorylation, maximal electron transfer capacity, complex 2 linked electron transfer and maximal complex 4 activity were analyzed using DatLab Software (version 7.4.0.4, Oroboros instruments corp., Innsbruck, Austria). The coupling efficiency was calculated by dividing complex 1 driven coupled respiration with complex 1 linked total respiration. Additionally, CS/CIV-ratio was calculated by dividing maximal citrate synthase (CS) activity with maximal complex 4 linked respiration, both per mg of tissue. Animals with greater residual oxygen consumption (ROX) than the respiration compensating for proton leak (LEAK) were excluded from data analysis due to possibly incomplete inhibition of complex-3. None of the control animals had >15% cytochrome-C response, indicating proper sample preparation[6]. No cancer animals were excluded due to >15% cytochrome-C response as this could be due to cancer or treatment. The final mitochondrial respirations were normalized in three different ways respectively; per tissue mass, citrate synthase activity and mitochondrial index measured using qPCR. Normalization per tissue mass revealing tissue level oxygen consumption rate and the citrate synthase activity and mitochondrial index normalization revealing oxygen consumption rate in relation to mitochondrial quantity assessed in different ways.

Table 1. The contents of biopsy preservation solution and mitochondrial respiration medium.

BIOPS	Mir05
2.77 mM CaK_2EGTA (100 mM stock)<br>7.33 mM K_2EGTA ((100 mM stock)<br>5.77 mM Na_2ATP (Sigma-Aldrich A2383)<br>20 mM Taurine (Acros Organics AC167111000T0625)<br>6.56 mM $MgCl_2$●6 H_2O (VWR 25108.295)<br>15 mM Na_2Phosphocreatine (EMD Millipore 2380)<br>20 mM Imidatzole (Merck 1.04716)<br>0.5 mM Dithiothreitol (MP Biomedicals 856126)<br>50 mM MES hydrate (Sigma-Aldrich M8250)	0.5 mM EGTA (Sigma-Aldrich E4378)<br>3 mM $MgCl_2$●6 H_2O (VWR 25108.295)<br>60 mM Lactobionic acid (Acros Organics AC167111000)<br>20 mM Taurine (Sigma-Aldrich T0625)<br>10 mM KH_2PO_4 (VWR 26936.260)<br>20 mM HEPES (Sigma-Aldrich H3375)<br>110 mM D-Sucrose (Sigma-Aldrich 84097)<br>1 g/l BSA, essentially fatty acid free (Sigma-Aldrich A6003)

The PH of BIOPS and Mir05 was adjusted to 7.1 at near 0˚C and near 30˚C respectively. BIOPS = biopsy preservation solution, Mir05 = mitochondrial respiration medium

Table 2. Mitochondrial respiration substrates and inhibitors and calculation of respiratory states.

Substrates and inhibitors in order of addition	Calculation of respiratory states
5 mM pyruvate (Acros Organics, cat. #132150250)<br>2 mM malate(Sigma-Aldrich M9138)<br>10 mM glutamate (Sigma-Aldrich G1626)	LEAK(-ROX)
5 mM ADP potassium salt with 0.6 mol MgCl2/mol ADP (Thermo Scientific, Alfa Aesar J60672)	CI-OXPHOS(-ROX-LEAK)
10 µM cytochrome C (Sigma-Aldrich C7752)	Cyt C response (increase of respiration, %)
10 mM succinate 10 (Sigma-Aldrich 14170)	CI&CII OXPHOS(-ROX,-LEAK)
titration of FCCP in 0.25 µM steps (Sigma-Aldrich C2920)	ETS max(-ROX)
0.5 µM rotenone (Sigma-Aldrich R-8875)	CII-ETS(-ROX)
2.5 µM antimycin A (Sigma-Aldrich A8674) + chamber re-oxygenation with H_2O_2 titration	ROX
2 mM ascorbate, 0.5 mM TMPD (Sigma-Aldrich 11140 & T3134)	CIV max(-ROX,-auto oxidation)
≥100 mM sodium azide (Sigma-Aldrich S-8032)	Autoxidation

CI-OXPHOS = complex 1 driven coupled respiration, CI&CII OXPHOS = complex 1 and 2 driven coupled respiration, CIV max = maximal activity of complex 4, CII-ETS = maximal electron transfer capacity of complex 2, Cyt C = cytochrome C, ETS max = maximal electron transfer capacity, LEAK = complex 1 driven uncoupled respiration, ROX = residual oxygen consumption.

Mitochondrial number determination with qPCR

Remaining mitochondrial respiration homogenate was snap frozen in liquid nitrogen and stored in -80˚C until the DNA extraction. The tissue homogenates were centrifuged at 16,000×*g* for 30 minutes at +4˚C to pellet all of the tissue and mitochondria. Most of the supernatant was removed from homogenates and universal salt extraction protocol by Aljanabi and Martinez (1997)[7] was used for DNA extraction, with addition of RNAase (Thermo Scientific™ EN0531) incubation step at +37˚C on shaker after proteinase K (Sigma-Aldrich P4850) incubation. The DNA concentrations and purity were determined with NanoDrop 2000 (Thermo Scientific), and the DNA integrity was confirmed with gel electrophoresis before the samples were aliquoted and stored at -80˚C until qPCR. For amplification of single copy nuclear gene of hexokinase 2 the following forward primer and reverse

primer were used respectively: 5'-TGCCACCCACTCGAAATACT-3', 5'-ACACCAGGGTAAAGGAGACG-3'. For the amplification of single copy mitochondrial gene of NADH-ubiquinone oxidoreductase chain 1 the following forward and reverse primers were used respectively: 5'-CTAGCAGAAACAAACCGGGC-3', 5'-CCGGCTGCGTATTCTACGTT-3'. The qPCR reactions were performed using 384-QuantStudioTM 12K Flex Real-Time PCR System (Thermo Fisher) with reaction volume of 5 µl with 15 ng and 0.075 ng of DNA for HK2 and ND1, respectively, and with final primer concentration of 1 µM and 2.5 µl of SensiFAST™ SYBR lo-ROX (Bioline). The qPCR reactions for the two genes from the same sample were run in triplicate on the same plate with plate containing DNA standard for determination of amplification efficiency and reference sample for determining gene copy number as well as inter-plate variability. The following qPCR conditions were used: pre-amplification 95˚C 5 min, amplification: 45 × (95˚C 10 s, 60˚C 10 s), melting curve: 95˚C 5 s, 66˚C 1 min, gradual increase to 97˚C.The intraplate coefficient of variation being <3% for both genes and the inter-plate variation being <2%. The mitochondrial copy-number index was determined with the following formula mtDNA = $2 \times 2^{\Delta Ct}$ where $\Delta Ct = C_T$(nDNA gene)- C_T(mDNA gene)[8].

Histology
A transversal tissue section (~3 mm) was cut from the middle of the mouse heart and fixed in 10% formalin for 48 hours and then transferred to 70% ethanol and stored at +4˚C until the dehydration and paraffin infiltration of tissues. The cardiac capillary vessels were stained in 5 µm tissue sections after deparaffinization with UltraClear (J.T.Baker, Phillipsburg, NJ, USA) and rehydrated with decreasing ethanol series using the Periodic Acid-Schiff (PAS) Stain used in our previous study[9] with 35-minute incubation in the Schiff's reagent (1.4 mM basic fuchsin; 0.1 M HCl; 2.4 mM sodium metabisulfite) and 10 minutes of color formation under running tap water. The stained sections were dehydrated and then sealed with DPX mounting media (Sigma-Aldrich, Merck, Darmstadt, Germany) and imaged with ×20 magnification using Nikon Eclipse Ni-E (upright), with a Nikon pE-300ultra camera and NIS-Elements AR-23 software. The LV capillary densities and cell number per randomly chosen areas were calculated manually, with experimenter blinded to the test groups, from minimum total area of ~4000 μm^2 using ImageJ software (version 1.53t, National Institutes of Health, USA).

Enzymatic assays and oxidative stress measurements
The mouse gastrocnemius muscle and remaining left ventricles were snap frozen in liquid nitrogen and stored in -80˚C until homogenization. The tissues were homogenized with a ratio of 1 mg of tissue per 10 µl of KF-Buffer (100 mM K-phosphate buffer, 150 mM KCl, pH 7.4) using two steel beads and the Bullet Blender® tissue homogenizer (Next Advance Inc., city, NY, USA). A Piece of left ventricle was also homogenized similarly in homogenizing solution (50 mM imidatzole, 1 mM EDTA, pH 7.2) for the measurement of 3-hydroxyacyl-CoA dehydrogenase (HOAD) activity. The raw homogenate in KF-buffer was aliquoted and diluted in 50 mM Tris pH 8.0 and 50 mM Tris pH 7.4 for citrate synthase activity (CS-activity) and lactate dehydrogenase activity (LDH-activity) measurement, respectively. The remaining KF-buffer raw homogenate was aliquoted for lipid peroxidation measurement (LPX) and for centrifugation at +4˚C 10,000×*g* for 15 minutes. The supernatant was aliquoted for the measurement of total protein content, protein carbonylation (CARB), superoxide dismutase activity (SOD), and catalase activity (CAT).

The HOAD-activity was measured from undiluted samples using kinetic spectrophotometry by pipetting samples in quadruplicate, first three wells receiving reaction solution (0.1 mM acetoacetyl CoA, 0.5 mM NADH, 50 mM imidatzole, 1 mM KCN, pH 7). The fourth well received reaction solution without acetoacetyl-CoA for measuring the background reactions using NADH. The

absorbance was measured at 349 nm in 37˚C for 30 times in 2.3 minutes to calculate reaction speed from the reaction slope. The CS-activity and LDH-activity measurement were done similarly using kinetic spectrophotometry and performed the same way as in our previous study Uurasmaa et al. (2021)[9]. The LPX-measurement was done by quantifying the lipid hydroxyl peroxides (LHPs) using the ferrous oxidation-xylenol orange (FOX) assay according to our previous study[9]. The total protein content was measured with a commercial bicinchoninic acid assay (Pierce, Thermo Scientific). CARB and SOD were also measured with commercial assays (Sigma-Aldrich 19160, MAK094). CARB-assay volumes were adjusted for measurement on 384-plate. SOD-activity was measured at 0.3 mg/ml of total protein and the assay was adjusted for 384-plate according to Stauffer et al. (2018)[10]. CAT-activity was measured at 0.6 mg/ml of total protein and the assay was performed according to Vuori & Kanerva (2018)[11].

All the assays were performed using multilabel plate reader (Perkin Elmer, EnSprire 2300). Outlier values >2×SD were excluded from analyses due to possibility of being not true physiological outliers but instead outliers introduced by methodology.

Statistical analysis

All the parameters obtained from one time point were tested for normality and the equality of variance using Shapiro-Wilk and Brown-Forsythe respectively. Single time point parameters were compared between four groups using two-way ANOVA to analyze the effects of chemotherapy and exercise within cancer groups (i.e. comparisons between T, TE, TD, TDE) and to analyze the effects of cancer and exercise within no-chemotherapy groups (i.e. comparisons between C, CE, T, TE). If data did not follow normal distribution LOG-transformation was performed, but original data was used for analysis if LOG-transformation did not improve data distribution or variance.

The running activity of exercise groups over time was compared using two-way RM ANOVA with time and group as factors. The other repeated measure parameters were compared between all the groups and time points using proc Glimmix linear mixed model on repeated measures with unstructured covariance structure to better account for missing data points and non-normally distributed data. First, all groups were compared together using exercise, and time point as well as chemotherapy nested under breast cancer as the factors. The interactions between these factors were also tested including the complex three-way interactions. Sufficient normality of residuals was confirmed with a histogram. The effect of cancer alone had to be tested separately comparing the groups without doxorubicin and this was done in the same way as previously but with the fixed effects of breast cancer, exercise, and time point.

For all group comparisons Holm-Sidak post-hoc test was performed if ANOVA or linear mixed model detected significant interactions between the factors. Post Hoc pairwise comparisons were performed between groups within time point and within groups between time points. Sigmaplot 15 was used for all the two-way ANOVA analyses and SAS® Enterprise Guide® was used for the linear model analysis. All figures were generated using SPSS 29 or Excel.

Spearman correlation analysis was performed separately for T2 and T3 between left ventricular glucose uptake and ejection fraction as well as left ventricular glucose uptake and left ventricular mass. Correlation of T3 EF with and mitochondrial coupling, LEAK and LV mass was also analyzed. Post hoc pairwise comparisons were performed between groups within time point and within groups between time points. The figures and the statistical testing were performed with Graph Pad Prism 5.01.

Supplemental Results as Figures and Tables

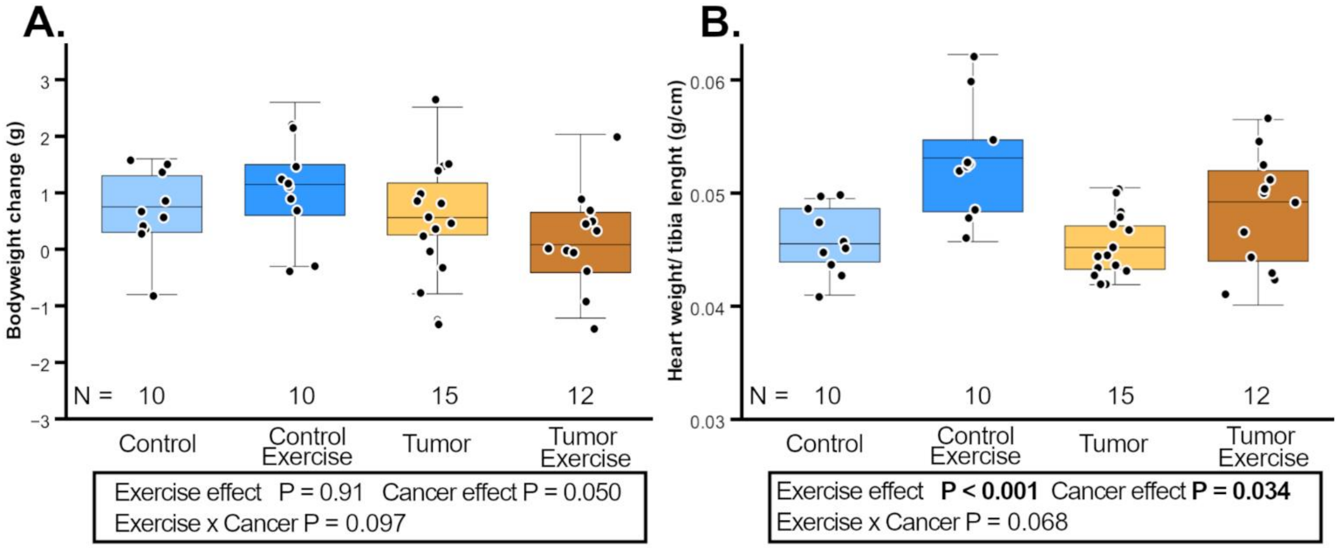

Figure 1. Bodyweights (A) and heart weights (B) in female FVB-mice with or without subcutaneous I3TC-tumors. Two-way ANOVA P-values <0.05 are highlighted in bold font and the panels have group n-numbers (N) indicated with numbers on the x-axis below each group boxplot. The data obtained for current manuscript in A and B from the control group and tumor group without exercise and without doxorubicin treatment has been previously published in Koivula et al. (2024) to support clinical findings[12].

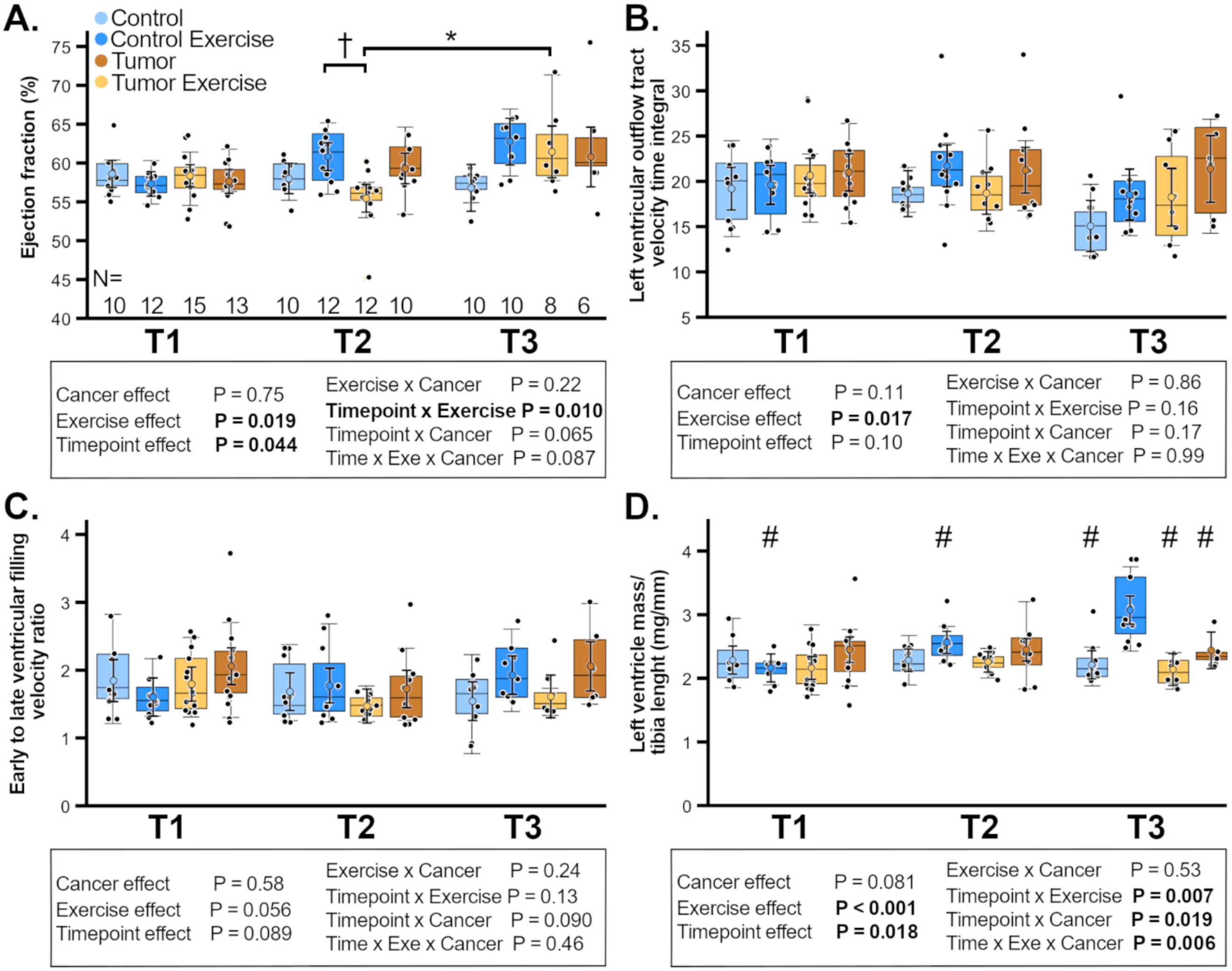

Figure 2. Changes in mouse cardiac function over time. Left ventricle cardiac ejection fraction (A), outflow track velocity time integral (B), early to late filling velocity (C) and mass to tibia length ratio (D) of female FVB-mice with or without subcutaneous I3TC-tumors at baseline (T1), 2.8 weeks (T2) and 4.8 weeks (T3) after tumor inoculation. Panel A has group n-numbers (N) indicated with numbers on the x-axis below each group boxplot. P-values <0.05 of repeated measure linear mixed model are highlighted in bold with model-predicted values±CI-95% shown with colored circle. Holm-Sidak: *P<0.05, †P<0.01 and #P<0.05 versus T3-Tumor-Exercise.

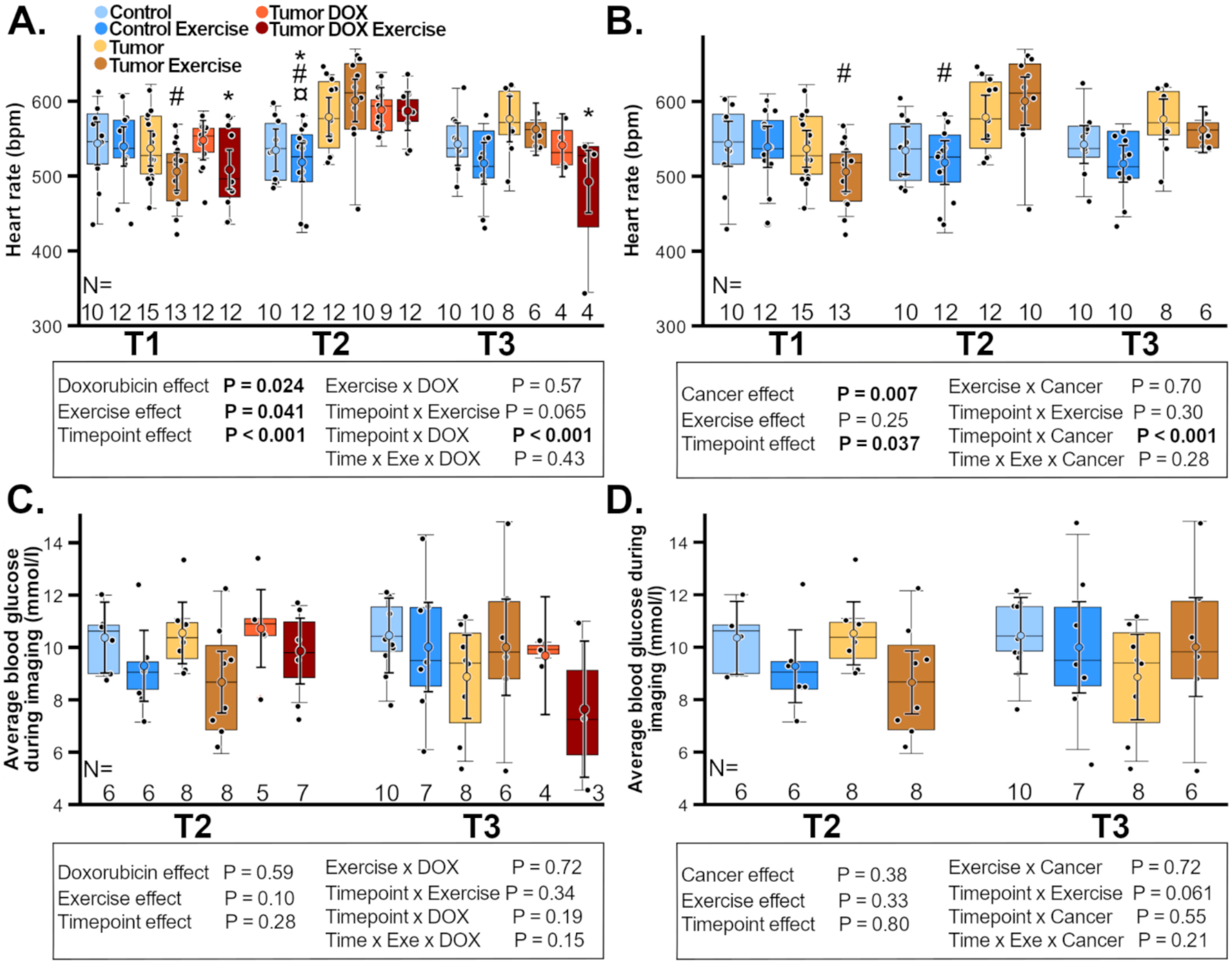

Figure 3. Mouse heart rate (A-B) and fasting blood glucose (C-D). Heart rate (HR) and blood glucose of female FVB-mice with or without subcutaneous I3TC-tumors and doxorubicin (DOX) treatment at baseline (T1), 2.8 weeks (T2) and 4.8 weeks (T3) after tumor inoculation. The panels have group n-numbers (N) indicated with numbers on the x-axis below each group boxplot. When all groups were compared the HR of all exercised tumor-bearing mice, with or without DOX treatment was increased at T2. Comparison of the no-DOX groups showed similarly that the HR was increased in T2 particularly in tumor exercise group. Linear mixed model on repeated measures P<0.05 are highlighted in bold with colored circles showing model-predicted values±CI-95%. Holm-Sidak: *P<0.05 vs T2-Tumor-DOX-Exercise, #P<0.05 vs T2-Tumor-Exercise, ¤P<0.05 vs T2-Tumor-DOX.

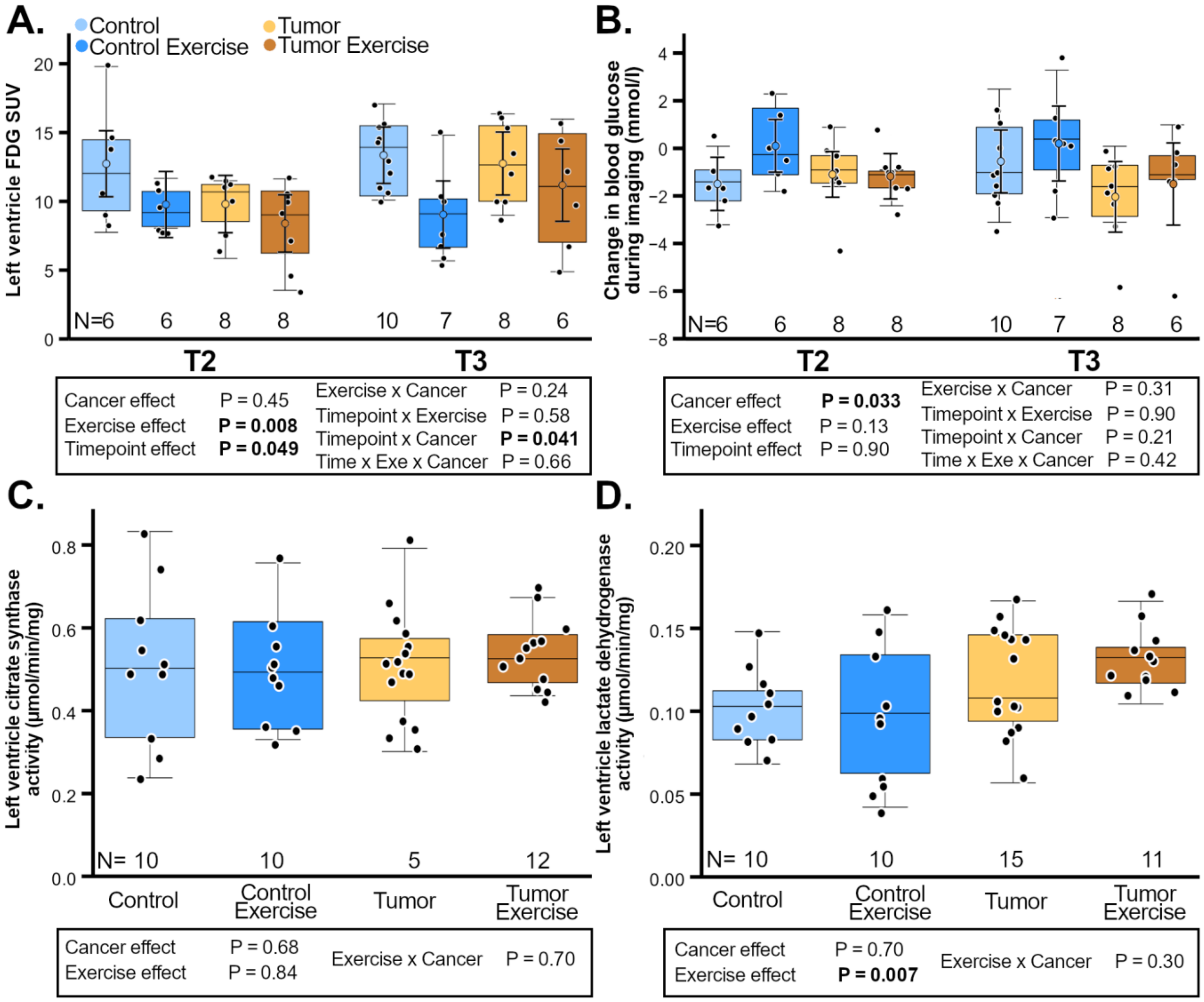

Figure 4. Heart glucose metabolism markers in female FVB-mice with/without subcutaneous I3TC-tumors. The left ventricle (LV) standardized FDG-uptake values (SUVs) indicative of LV glucose uptake (A) and the change in blood glucose levels during PET-imaging (B) are shown for 2.8 weeks (T2) and 4.8 weeks (T3) after tumor inoculation and the enzyme function at euthanasia (C-D). The panels have group n-numbers (N) indicated with numbers on the x-axis below each group boxplot. P <0.05 of linear mixed model on repeated measures (A-B) or two-way ANOVA (C-D) are highlighted in bold. Colored circles are showing linear mixed model-predicted values±CI-95% (A-B).

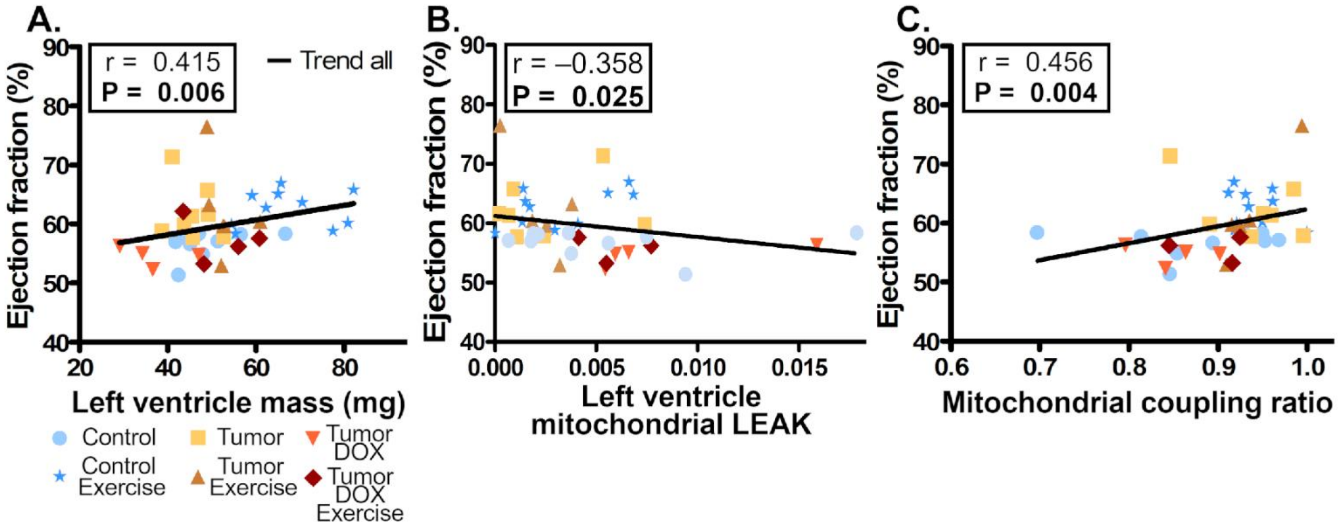

Figure 5. Ejection fraction correlation with left ventricle mass and mitochondrial function. The correlations are shown at T3 for left ventricle mass (A), mitochondrial proton leak (B) and mitochondrial coupling ratio (C) in female FVB-mice with or without subcutaneous I3TC-tumors or doxorubicin treatment. Spearman-correlation P<0.05 are highlighted in bold. N-numbers for control, control exercise, tumor, tumor exercise, tumor DOX and Tumor DOX exercise are 10, 10, 7, 6, 4 and 4 respectively.

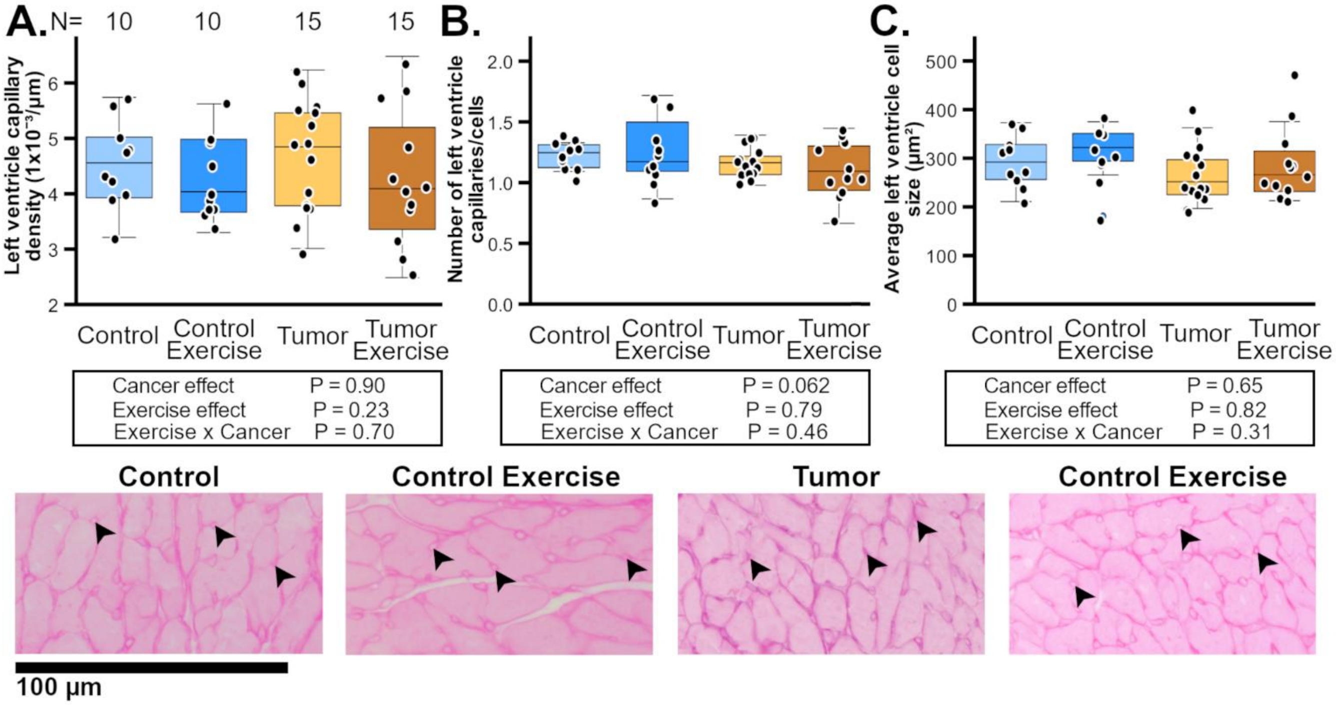

Figure 6. Heart histology in female FVB-mice with or without subcutaneous I3TC-tumors. Left ventricle capillary density (A), number of capillaries per cells (B) and transversal cell area (C) are shown with exemplary PAS-stained histological 20× images with contrasts and tone adjusted for visibility. Arrowheads denote the capillary vessels. Panel A has group n-numbers (N) indicated with numbers above each group boxplot. The data obtained for current manuscript in A-C from the control group and tumor group without exercise and without doxorubicin treatment has been previously published in Koivula et al. (2024) to support clinical findings[12].

Table 3. Doxorubicin and exercise effect on gastrocnemius weight, oxidative stress, and metabolic enzyme activities.

	Tumor	Tumor Exercise	Tumor DOX	Tumor DOX Exercise	DOX effect	Exercise effect	DOX × Exercise
G-weight /tibia length (g/cm)	0.054±0.004	0.054±0.006	0.050±0.003	0.050±0.005	**0.005**	0.87	0.99
CS-activity (µmol/min/mg)	0.030±0.010	0.037±0.014	0.026±0.006	0.039±0.018	0.84	**0.007**	0.44
LDH-activity (µmol/min/mg)	0.667±0.183	0.679±0.096	0.640±0.105	0.650±0.170	0.51	0.80	0.98
LPX-level (µM/mg)	4.269±2.053	5.797±1.950	4.224±1.592	5.525±1.357	0.76	**0.009**	0.83
N-Number	15, $14^{CS,\ LPX}$	12, $11^{LDH,\ LPX}$	12, $11^{LDH,\ CS}$	12, 11^{LDH}			

Groups consist of subcutaneous I3TC-tumor bearing female FVB-mice with or without doxorubicin treatment. If not stated otherwise all the values are presented relative to protein amount and are shown as mean±SD with two-way ANOVA tests with P<0.05 highlighted in bold. N-numbers apply to all assays unless stated otherwise with superscript.CS = citrate synthase, DOX = doxorubicin, LDH = lactate dehydrogenase, LPX = lipid peroxidation indicated by lipid hydroxyl peroxides.

Table 4. Cancer and exercise effect on gastrocnemius weight, oxidative stress, and metabolic enzyme activities.

	Control	Control Exercise	Tumor	Tumor Exercise	Cancer effect	Exercise effect	Cancer × Exercise
Gastrocnemius weight /tibia length (g/cm)	0.055±0.005	0.057±0.003	0.054±0.004	0.054±0.006	0.14	0.31	0.4
CS-activity (µmol/min/mg)	0.026±0.011	0.042±0.012	0.030±0.010	0.037±0.014	0.89	**0.002**	0.24
LDH-activity (µmol/min/mg)	0.805±0.204	0.707±0.101	0.667±0.183	0.679±0.096	0.09	0.37	0.26
LPX-level (µM/mg)	3.770±1.415	4.728±3.093	4.269±2.053	5.797±1.950	0.07	0.25	0.68
N-Number	10, 9^{LPX}	10, 9^{LDH}	15, $14^{CS,\ LPX}$	12, $11^{LDH,\ LPX}$			

Groups consist of female FVB-mice with or without subcutaneous I3TC-tumors without DOX treatment. If not stated otherwise all the values are presented relative to protein amount and are shown as mean±SD with two-way ANOVA P<0.05 highlighted in bold. N-numbers apply to all assays unless stated otherwise with superscript. CS = citrate synthase, LDH = lactate dehydrogenase, LPX = lipid peroxidation indicated by lipid hydroxyl peroxides.

Table 5. Cancer and exercise effect on left ventricle mitochondrial respiration parameters.

	Control N = 10	Control Exercise N = 10	Tumor N = 10	Tumor Exercise N = 8	Cancer effect	Exercise effect	Cancer × Exercise
LEAK (pmol/(s×mDNA)) (pmol/(s×mg)) (pmol/(s×CS))	0.005±0.005<br>23.9±20.5<br>8.7±7.6	0.003±0.002<br>13.5±9.3<br>5.1±4.1	0.004±0.003<br>14.1±11.9<br>4.5±3.2	0.003±0.002<br>12.8±6.7<br>4.0±2.1	0.43<br>0.24<br>0.10	0.15<br>0.20<br>0.19	0.64<br>0.31<br>0.33
CI-OXPHOS (pmol/(s×mDNA)) (pmol/(s×mg)) (pmol/(s×CS))	0.036±0.010<br>167.2±29.2<br>64.0±32.1	0.049±0.022<br>203.9±71.2<br>72.8±42.4	0.041±0.016<br>141.6±43.6<br>47.9±8.0	0.037±0.012<br>165.3±38.2<br>51.0±12.0	0.54<br>0.05<br>0.07	0.39<br>0.07<br>0.53	0.12<br>0.68<br>0.83
CI&CII-OXPHOS (pmol/(s×mDNA)) (pmol/(s×mg)) (pmol/(s×CS))	0.11±0.02<br>497.6±67.0<br>184.5±69.1	0.13±0.05<br>555.2±174.3<br>194.8±92.5	0.14±0.03<br>492.6±96.3<br>180.0±66.8	0.12±0.04<br>518.4±97.19<br>160.8±35.3	0.42<br>0.70<br>0.57	0.93<br>0.44<br>0.77	0.12<br>0.91<br>0.68
ETS max (pmol/(s×mDNA)) (pmol/(s×mg)) (pmol/(s×CS))	0.11±0.03<br>532.7±75.2<br>197.0±71.9	0.14±0.05<br>575.4±173.4<br>201.9±91.9	0.14±0.03<br>513.2±101.0<br>186.9±66.6	0.12±0.05<br>534.4±101.7<br>165.6±35.8	0.52<br>0.52<br>0.32	0.80<br>0.59<br>0.73	0.14<br>0.99<br>0.58
CII-ETS (pmol/(s×mDNA)) (pmol/(s×mg)) (pmol/(s×CS))	0.08±0.02<br>390.1±68.3<br>143.3±50.7	0.09±0.04<br>400.7±127.6<br>140.8±67.4	0.11±0.02<br>390.1±88.1<br>144.0±61.2	0.09±0.04<br>398.9±75.5<br>123.8±28.1	0.28<br>0.98<br>0.80	0.60<br>0.75<br>0.49	0.22<br>0.98<br>0.88
CIV max (pmol/(s×mDNA)) (pmol/(s×mg)) (pmol/(s×CS))	0.29±0.09<br>1353.3±286.0<br>525.6±292.4	0.33±0.09<br>1417.2±364.7<br>475.2±140.9	0.39±0.10<br>1393.3±257.5<br>507.4±169.1	0.38±0.2<br>1601.2±547.6<br>491.1±153.2	0.07<br>0.36<br>0.82	0.77<br>0.27<br>0.85	0.53<br>0.55<br>0.97
Cyt C response (%)	6.6±4.9	7.2±5.8	11.7±12.0	10.7±7.4	0.11	0.93	0.77
Coupling efficiency	0.88±0.08	0.94±0.03	0.92±0.06	0.93±0.03	0.56	0.06	0.23
(CS-activity/CIV) $\times 10^{-3}$	0.41±0.20	0.36±0.06	0.39±0.11	0.39±0.12	0.72	0.92	0.72
Mitochondrial index	2471.4±683	2235.6±563	1823.8±301	2490.3±1359	0.45	0.40	0.09

Groups consist of female FVB-mice with or without subcutaneous I3TC-tumors without DOX treatment. All the values shown are mean±SD normalized to mitochondrial index, tissue weight and citrate synthase activity respectively, unless stated otherwise, with two-way ANOVA P-values shown. CI-OXPHOS = Complex 1 linked oxidative phosphorylation CI&CII-OXPHOS = Complex 1 & Complex 2 linked oxidative phosphorylation, CII-ETS = Complex 2 linked electron transfer capacity, CIV max = maximal activity of complex 4, CS = citrate synthase, Cyt C = cytochrome C, DOX = Doxorubicin, ETS max = maximal electron transfer capacity, LEAK = complex 1 driven uncoupled respiration.

Table 6. Cancer and exercise effect on left ventricle oxidative stress, anti-oxidative enzymes, and metabolic enzyme activities.

	Control	Control Exercise	Tumor	Tumor Exercise	Cancer effect	Exercise effect	Cancer × Exercise
HOAD (µmol/min/mg)	0.13±0.11	0.10±0.08	0.08±0.04	0.10±0.04	0.25	0.88	0.33
SOD-activity (%)	69.6±4.1	70.0±3.2	68.8±4.4	68.3±3.5	0.30	0.97	0.70
CAT-activity (µmol/min/mg)	35.9±2.7	35.0±5.2	34.6±5.4	38.2±6.7	0.55	0.41	0.18
LPX-level (µM/mg)	38.7±6.4	29.3±5.3	36.4±5.4	37.9±5.6	0.27	0.52	0.78
CARB-level (µmol/mg)	11.3±2.0	11.6±2.2	11.5±2.1	11.5±2.2	0.91	0.83	0.84
N-Number	10, 9^{CAT}	10, $9^{CARB, SOD}$	15, 14^{HOAD}	12, 11^{SOD}			

Groups consist of female FVB-mice with or without subcutaneous I3TC-tumors without DOX treatment. All the values are shown mean±SD per protein content unless stated otherwise with two-way ANOVA P-values. N-numbers apply to all assays unless stated otherwise in superscript. CAT = catalase, CARB = protein carbonylation, DOX = doxorubicin, HOAD = 3-hydroxyacyl-CoA dehydrogenase, LPX = lipid peroxidation indicated by lipid hydroxyl peroxides, SOD = superoxide dismutase.

References

1 Naaktgeboren WR, Binyam D, Stuiver MM et al. Efficacy of Physical Exercise to Offset Anthracycline-Induced Cardiotoxicity: A Systematic Review and Meta-Analysis of Clinical and Preclinical Studies. J Am Heart Assoc. 2021;10:e021580.

2 Weiland A, Roswall P, Hatzihristidis TC, Pietras K, Ostman A, Strell C. Fibroblast-dependent regulation of the stem cell properties of cancer cells. Neoplasma. 2012;59:719–727.

3 Rundqvist H, Veliça P, Barbieri L et al. Cytotoxic T-cells mediate exercise-induced reductions in tumor growth. Elife. 2020;9:e59996.

4 Rainio O, Han C, Teuho J et al. Carimas: An Extensive Medical Imaging Data Processing Tool for Research. J Digit Imaging. 2023;36:1885–1893.

5 Cantó C, Garcia-Roves PM. High-Resolution Respirometry for Mitochondrial Characterization of Ex Vivo Mouse Tissues. Curr Protoc Mouse Biol. 2015;5:135–153.

6 Kuznetsov A V, Veksler V, Gellerich FN, Saks V, Margreiter R, Kunz WS. Analysis of mitochondrial function in situ in permeabilized muscle fibers, tissues and cells. Nat Protoc. 2008;3:965–976.

7 Aljanabi S. Universal and rapid salt-extraction of high quality genomic DNA for PCR- based techniques. Nucleic Acids Res 1997;25:4692–4693.

8 Quiros PM, Goyal A, Jha P, Auwerx J. Analysis of mtDNA/nDNA Ratio in Mice. Curr Protoc Mouse Biol 2017; 7: 47–54.

9 Uurasmaa T-M, Streng T, Alkio M, Heinonen I, Anttila K. Short-term exercise affects cardiac function ex vivo partially via changes in calcium channel levels, without influencing hypoxia sensitivity. J Physiol Biochem. 2021;77:639–651.

10 Stauffer J, Panda B, Ilmonen P. Telomere length, sibling competition and development of antioxidant defense in wild house mice. Mech Ageing Dev. 2018;169:45–52.

11 Vuori K, Kanerva M. Catalase (CAT) activity assay for zooplankton samples. protocols.io 2018.

12 Koivula T, Uurasmaa T-M, Han C et al. Myocardial blood flow in newly diagnosed breast cancer patients at rest and during exercise. iScience 2024;**27**:111081.
